# Systematic Nanoscale Analysis of Endocytosis Links Efficient Vesicle Formation to Patterned Actin Nucleation

**DOI:** 10.1016/j.cell.2018.06.032

**Published:** 2018-08-09

**Authors:** Markus Mund, Johannes Albertus van der Beek, Joran Deschamps, Serge Dmitrieff, Philipp Hoess, Jooske Louise Monster, Andrea Picco, François Nédélec, Marko Kaksonen, Jonas Ries

**Affiliations:** 1 Cell Biology and Biophysics Unit, European Molecular Biology Laboratory (EMBL), Meyerhofstrasse 1, 69117 Heidelberg, Germany; 2 Department of Biochemistry and NCCR Chemical Biology, University of Geneva, Quai Ernest Ansermet 30, 1211 Geneva, Switzerland; 3 Collaboration for joint PhD degree between EMBL and Heidelberg University, Faculty of Biosciences

**Keywords:** endocytosis, actin, WASP, clathrin, high-throughput, superresolution microscopy, single-molecule localization microscopy, SMLM, Brownian dynamics simulations

## Abstract

Clathrin-mediated endocytosis is an essential cellular function in all eukaryotes that is driven by a self-assembled macromolecular machine of over 50 different proteins in tens to hundreds of copies. How these proteins are organized to produce endocytic vesicles with high precision and efficiency is not understood. Here, we developed high-throughput superresolution microscopy to reconstruct the nanoscale structural organization of 23 endocytic proteins from over 100,000 endocytic sites in yeast. We found that proteins assemble by radially ordered recruitment according to function. WASP family proteins form a circular nanoscale template on the membrane to spatially control actin nucleation during vesicle formation. Mathematical modeling of actin polymerization showed that this WASP nano-template optimizes force generation for membrane invagination and substantially increases the efficiency of endocytosis. Such nanoscale pre-patterning of actin nucleation may represent a general design principle for directional force generation in membrane remodeling processes such as during cell migration and division.

## Highlights

• High-throughput superresolution imaging of 23 proteins at thousands of endocytic sites
• Endocytic proteins organize into nanoscale radial zones according to their function
• A circular WASP nano-template patterns actin filament nucleation
• The WASP nano-template is crucial for high efficiency of endocytosis

## Graphical Abstract

**Figure:**
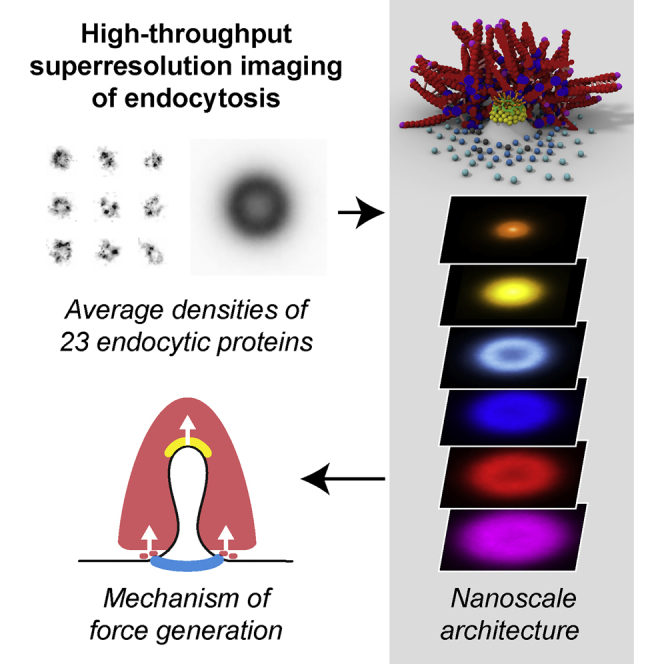

## Teaser

Superresolution analysis of endocytic vesicle assembly identifies a patterned actin template that orchestrates protein recruitment.

## Introduction

Clathrin-mediated endocytosis (CME) is critical for many biological processes such as signaling, nutrient uptake, and pathogen entry and involves the internalization of cargo molecules from the cell surface into small membrane vesicles. CME follows a stereotypic order of events: first, a protein coat assembles on the membrane, which then invaginates to form a vesicle with cargo molecules inside. This vesicle is pinched off the plasma membrane and rapidly uncoats allowing fusion with endosomes. CME is performed by a machinery that comprises more than 50 different proteins and is conserved from yeast to humans. Much of our knowledge of the mechanism of CME comes from research in cultured mammalian cells, and yeast (Kaksonen and Roux, 2018, McMahon and Boucrot, 2011, Weinberg and Drubin, 2012). In the budding yeast *Saccharomyces cerevisiae*, genetic and imaging screens have led to a near-complete parts list (Weinberg and Drubin, 2012). In addition, live-cell imaging has revealed the order of assembly of components and categorized them into modules based on their dynamics (Kaksonen et al., 2003, Kaksonen et al., 2005). This modular organization is remarkably conserved in metazoans (Boettner et al., 2011, Kaksonen and Roux, 2018, McMahon and Boucrot, 2011, Weinberg and Drubin, 2012).

In yeast, CME can be divided into an early phase, when endocytic proteins are recruited to a flat membrane (Kukulski et al., 2012), and a late phase during which invagination occurs. The early phase is characterized by the recruitment of various endocytic adaptor and coat proteins, including clathrin, and is long and variable in duration (Kaksonen et al., 2005, Stimpson et al., 2009). The following highly regular late phase begins with the arrival of late coat proteins, followed by actin regulatory proteins including WASP and type I myosins (Sun et al., 2006). A burst of actin polymerization starts membrane invagination, and with the arrival of amphiphysin proteins, vesicle scission occurs (Picco et al., 2015).

Although the recruitment timing of proteins during endocytosis is well understood, their spatial organization at endocytic sites is largely unknown. This is due to the complexity, dynamics, and small size of the endocytic machinery, which is below the resolution of conventional light microscopy. Live-cell fluorescence microscopy revealed the average positions of endocytic proteins along the membrane invagination (Berro and Pollard, 2014a, Picco et al., 2015) and the shape of the invagination was determined by correlative light and electron microscopy (CLEM) (Kukulski et al., 2012). Furthermore, immuno-electron microscopy (EM) reported the approximate location of some endocytic proteins (Idrissi et al., 2008, Idrissi et al., 2012) in the late stages of endocytosis. However, systematic information about the location of the different endocytic proteins is currently lacking, particularly during the initial phase before membrane bending. Thus, the molecular architecture of this complex supramolecular machine, and how it can drive endocytosis so efficiently to generate precisely shaped and sized membrane vesicles, remains unknown.

To address this fundamental gap in our knowledge, we developed a high-throughput superresolution microscopy pipeline to study the nanoscale organization of proteins in the endocytic machinery over the entire endocytic time line. By automating image acquisition and analysis, we could process superresolution images of thousands of cells. We used budding yeast to enable systematic fluorescent tagging of endocytic proteins at their genomic loci to ensure high labeling efficiency, while retaining native expression and biological function. This approach allowed us to analyze the structural organization of 23 different endocytic proteins sampled throughout the endocytic process at, in total, over 100,000 endocytic sites in more than 20,000 fixed yeast cells.

We found that assembly of the machinery initiates stochastically from irregular structures. Thereafter, an intricate self-organization emerges where endocytic proteins are radially ordered according to their function. WASP family proteins formed a ring-shaped nanoscale template on the flat membrane to pattern the nucleation of actin filaments. Brownian dynamics simulations of actin polymerization showed that this geometry enables the formation of a scaffold of actin filaments producing sufficient force for membrane invagination, and centering provides a mechanism for the high efficiency and robustness of vesicle budding.

## Results

### Experimental Pipeline

**Figure 1:**
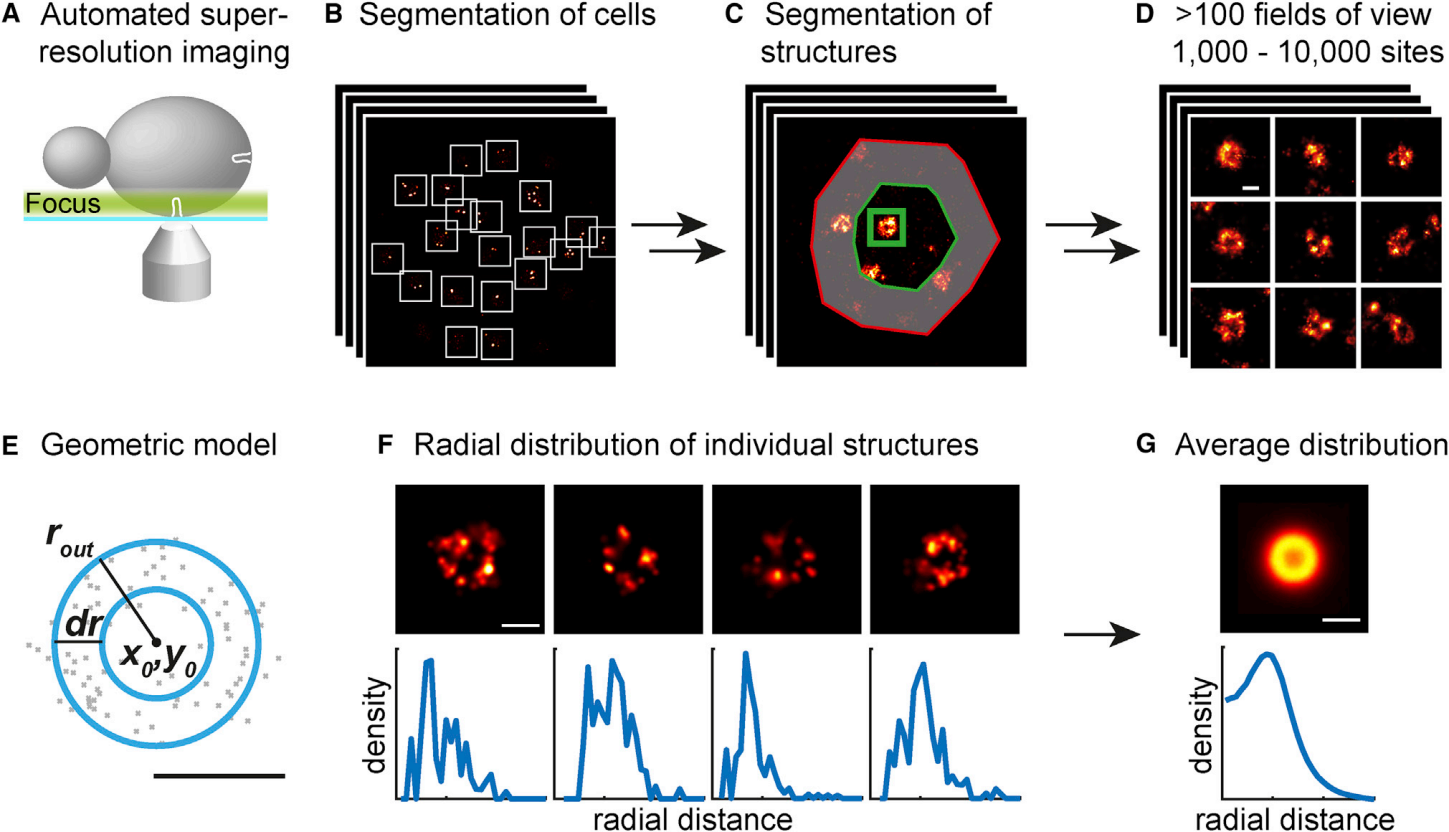
High-Throughput Superresolution Imaging of Endocytosis in Yeast (A) Fixed yeast cells expressing fluorescently tagged endocytic proteins were imaged using 2D high-throughput superresolution microscopy with the focal plane at the bottom of the cells. Images contain the 2D projection of the entire endocytic site in the membrane plane. (B–E) Cells (B) and endocytic sites were automatically segmented only in the center of cells (C) to avoid tilted structures. Individual endocytic sites (D) were analyzed by fitting a single geometric model (E) to determine center coordinates *x*_*0*_*, y*_*0*_, outer radius *r*_*out*_, and a rim with a thickness *dr*. The model accounts for the localization precision and describes both patch-like (*dr ≥ r*_*out*_) and ring-like (*dr < r*_*out*_) structures. (F) The radial density distribution around *x*_*0*_*, y*_*0*_ was calculated for each site. (G) Using *x*_*0*_*, y*_*0*_ individual sites were aligned by translation, and the average protein distribution and radial density profiles were calculated. Scale bars represent 100 nm. See also Figures S1, S2, and S3 and Table S1.

**Figure S1:**
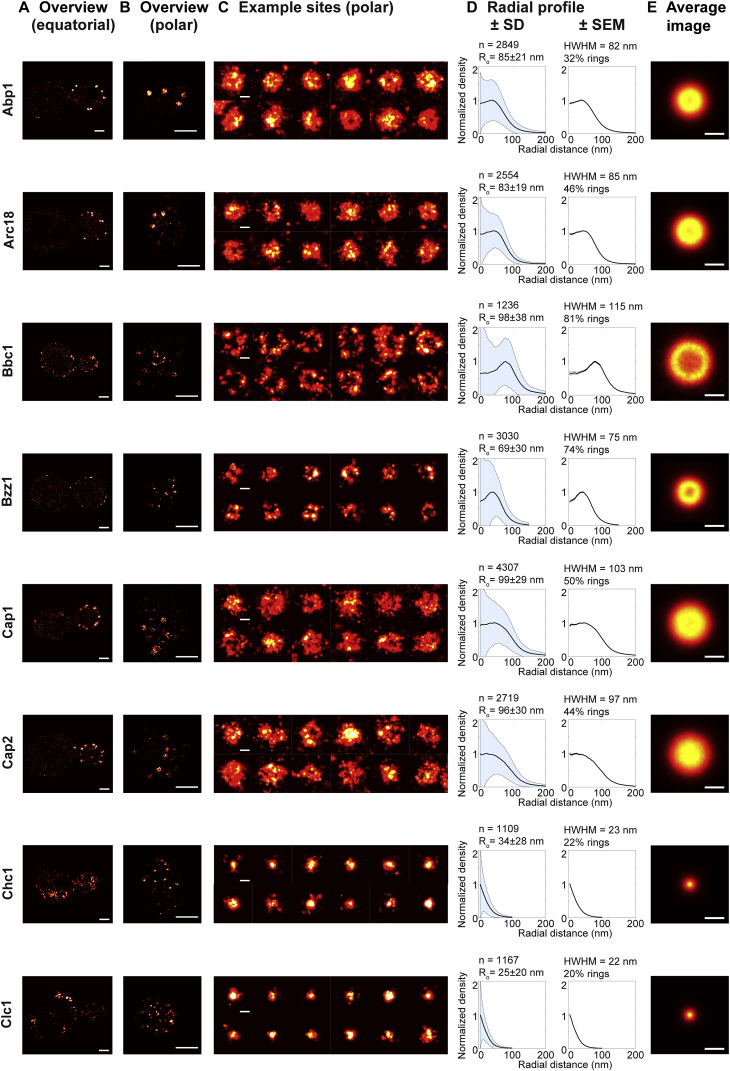
Overview of Imaged Endocytic Proteins (Part 1/3), Related to Figures 1 and 2 (A and B) Shown are superresolved images of cells where the focal plane was positioned on the midplane (A) and bottom (B) of the cells. (C) Shows example endocytic sites focused as in (B). (D) Shows average radial profiles. Shaded areas correspond to the standard deviation (left) or standard error of the mean (right). (E) Shows the average image. The number of sites, fraction of rings as obtained by the fit from the *dr/r*_*out*_ values (see the STAR Methods for details), the half-maximum of radial profiles (HWHM), as well as the mean and standard deviation of the outer radius as obtained by the fit are indicated. Scale bars 1 μm (A and B) or 100 nm (C and E).

**Figure S2:**
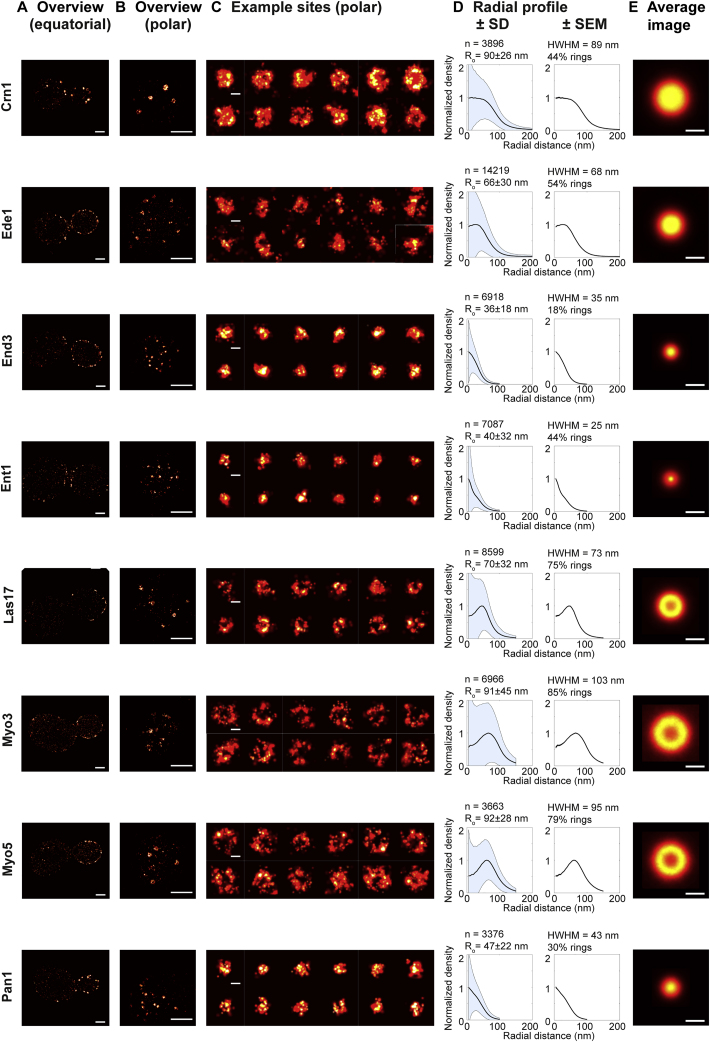
Overview of Imaged Endocytic Proteins (Part 2/3), Related to Figures 1 and 2 (A–E) As in Figure S1. Scale bars 1 μm (A and B) or 100 nm (C and E).

**Figure S3:**
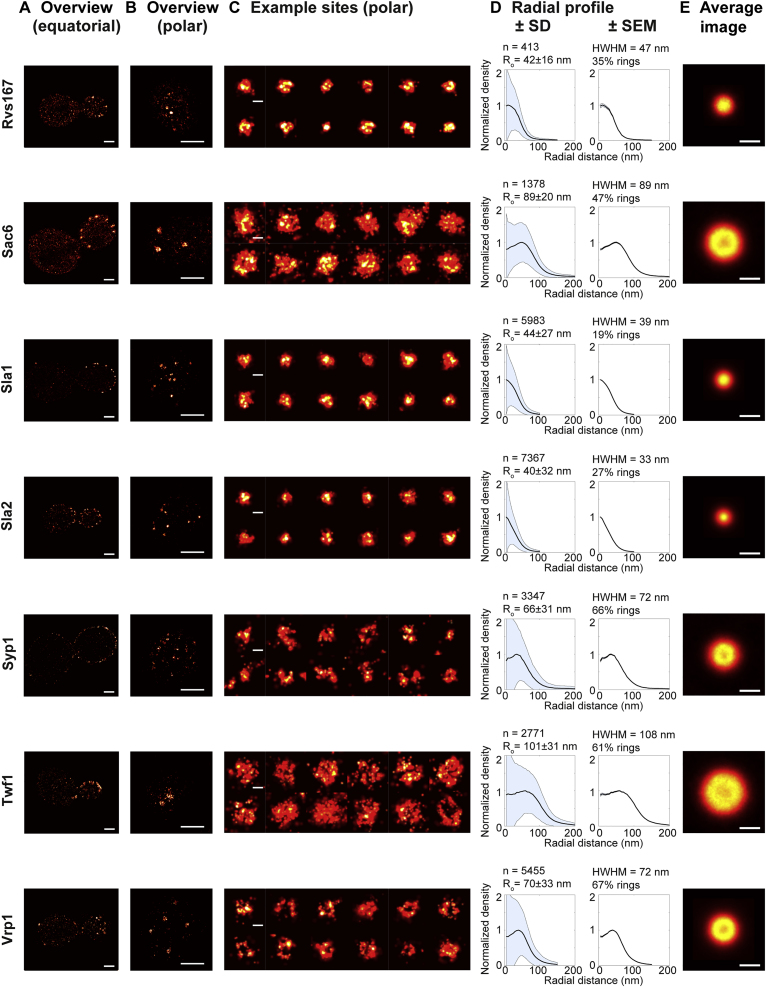
Overview of Imaged Endocytic Proteins (Part 3/3), Related to Figures 1 and 2 (A–E) As in Figure S1. Scale bars 1 μm (A and B) or 100 nm (C and E).

Here, we used single-molecule localization microscopy (SMLM, also called “(f)PALM” or “STORM”) (Betzig et al., 2006, Hess et al., 2006, Rust et al., 2006) to image sites of clathrin-mediated endocytosis in budding yeast strains with single endocytic proteins endogenously tagged at their C termini with a photoswitchable fluorescent protein. We fixed the cells with formaldehyde, and then placed the focal plane on their underside, where endocytic invaginations are oriented perpendicularly to the focal plane (Figure 1A). Thereby we obtained two-dimensional (2D) projections of endocytic structures, which reveal the lateral distribution of proteins at endocytic sites. In these images, the distribution of endocytic proteins appeared as patches, rings, or irregular shapes (Figures S1, S2, and S3).

Because endocytosis was arrested by fixation, the individual images provide snapshots of different endocytic time points. To sample the entire endocytic timeline with high statistical power, we automatically acquired and segmented superresolution images of many thousands of endocytic sites (Figures 1B–1D), quantitatively analyzed individual structures (Figures 1E and 1F), spatially aligned them by translation, and averaged them. We thereby generated density profiles of how each protein is on average distributed around the center of the endocytic site (Figure 1G), representing the average structural organization of endocytic proteins over their lifetime. Additionally, we determined how the distribution of four key endocytic proteins evolves during endocytosis.

### The Functional Modules of Endocytosis Occupy Distinct Radial Zones

**Figure 2:**
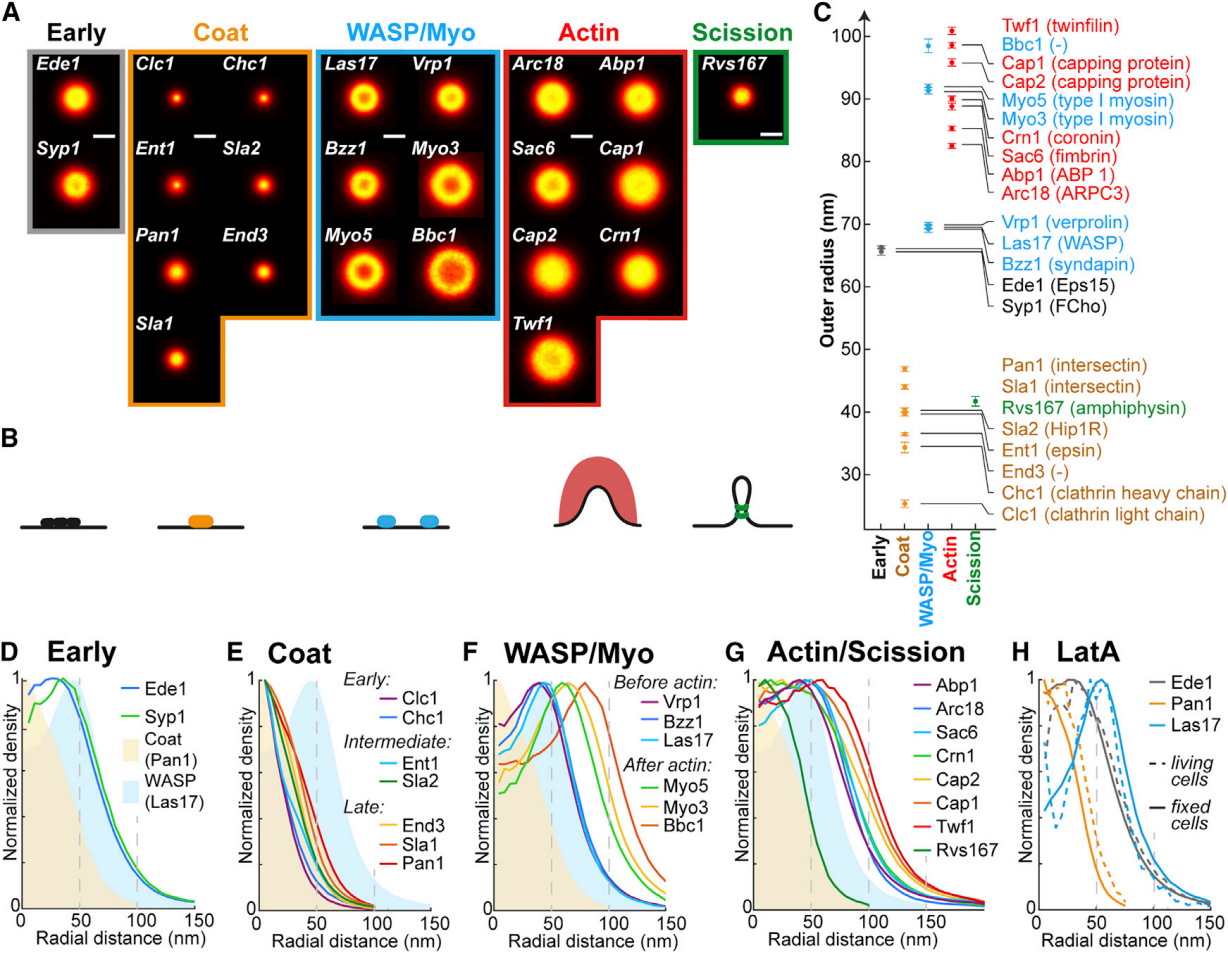
Average Radial Distribution of 23 Proteins in the Endocytic Machinery (A) Endocytic proteins form very diverse structures. Shown are the average images for 23 endocytic proteins (for a description, see text). (B) Side-view schematic of structures formed by the endocytic modules on the plasma membrane. (C) Outer radii of structures of 23 endocytic proteins (mean ± SEM; number of sites and SD in Table S1). Mammalian homologs in parentheses, as identified in Weinberg and Drubin (2012). (D–G) Average radial profiles of endocytic proteins in the early (D), coat (E), WASP (F), and actin (G) modules. Shaded areas indicate the endocytic coat (beige, profile of Pan1) and WASP modules (blue, profile of Las17). (H) Average radial profiles of Ede1, Pan1, and Las17 after LatA treatment, analyzed both in living and in fixed cells. Scale bars represent 100 nm. See Figures S1, S2, S3, and S4 for representative images of all proteins.

We determined the structural organization of 23 endocytic proteins from all functional modules of the machinery (Figures 2A, S1, S2, and S3, mammalian homologs in parentheses): the early proteins Ede1 (Eps15) and Syp1 (an F-BAR protein, FCHo), which initiate endocytic sites; the seven coat proteins Clc1 and Chc1 (clathrin light and heavy chain), Sla2 (Hip1R), Ent1 (epsin), Sla1, End3, and Pan1 (all intersectin), which interact with membrane, cargo, and actin; six WASP/Myo module proteins including the actin nucleation promoting factor Las17 (WASP), Vrp1 (verprolin), the F-BAR protein Bzz1 (syndapin), the type-I myosins Myo3 and Myo5 (both Myo1-E), and Bbc1 (fungi only); seven components of the endocytic actin network including the Arp2/3 complex subunit Arc18 (ArpC3), capping proteins Cap1 and Cap2 (Capping protein), actin binding protein Abp1 (ABP1), actin crosslinker Sac6 (fimbrin), actin turnover factors Twf1 (twinfilin) and Crn1 (coronin); and, finally, the N-BAR protein Rvs167 (amphiphysin). For each of these proteins, we acquired superresolution images of ∼1,000–10,000 endocytic sites (except Rvs167, where n = 413) (Table S1).

These images revealed how proteins are arranged at endocytic sites: Some proteins, including the coat proteins, formed mostly small patches (Figures S1, S2, and S3). Others, like WASP/Myo proteins, formed mostly ring-like structures with empty centers (Figures S1, S2, and S3). For all proteins, except Ede1 and Syp1, these structures were characteristic in size and in shape, indicating an intricate sub-organization of different components within the endocytic machinery.

Next, we quantitatively analyzed the individual superresolution images of all structures using a geometric model (Figures 1E and 1F) to extract their shape, size, and position, derived each protein’s average distribution and radial density profile (Figures 2D–2G, S1, S2, and S3), and classified the shape of the average protein distribution as a patch, ring or dome (Table S1). We found the average distribution of a protein (Figure 2A) to be representative of the individual structures (Figures S1, S2, and S3), but note that specific angular distributions or structural asymmetry that are visible in individual images are not represented in the averages. By design, the radial density profiles (Figures 2D–2G) represent the average probability of an endocytic protein to be found at a certain distance from the center of the endocytic site and are intended to quantitatively compare the distribution of different proteins.

These data showed that endocytic proteins assemble into nanoscale structures of different sizes and geometries, as evident both in images of individual sites (Figures S1, S2, and S3) and in the averages (Figure 2). Proteins within the same functional endocytic module had similar radial profiles and sizes, indicating that structural organization and function are linked. Coat proteins formed the smallest structures, which were mostly patches with mean outer radii of ∼25–50 nm. Proteins of the WASP/Myo module formed larger, ring-like structures with outer radii of ∼60–100 nm, and actin module proteins formed the largest structures with outer radii of ∼80–100 nm. The amphiphysin Rvs167 of the scission module formed small structures with an outer radius of ∼40 nm. Together, our results reveal that endocytic proteins are functionally organized in distinct radial zones at endocytic sites.

**Figure S4:**
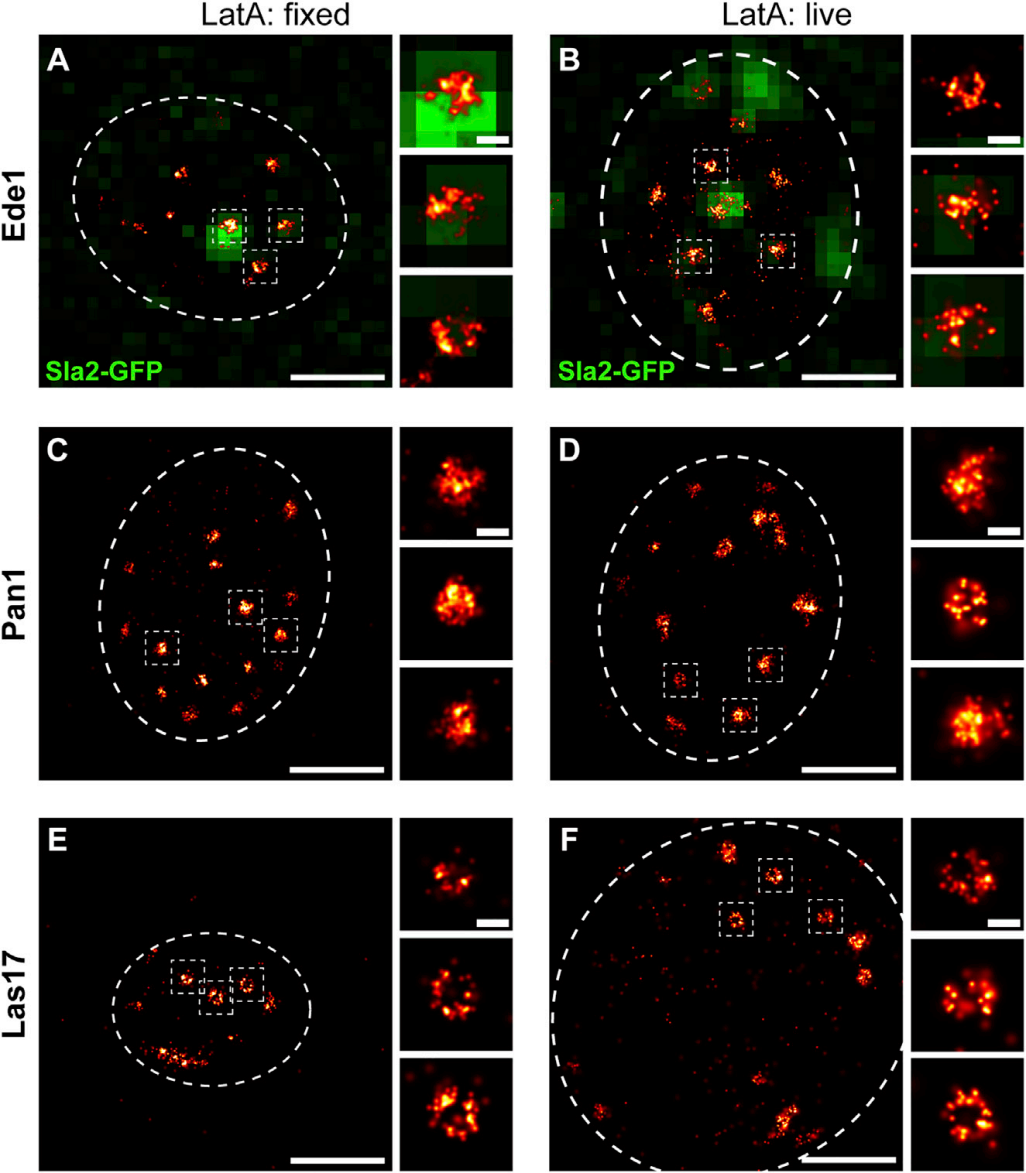
Ede1, Pan1, and Las17 Structures after Latrunculin A Treatment, Related to Figure 2H (A–F) Shown are images of fixed (A, C, and E) and living (B, D, and F) cells, where endocytic sites have been arrested on flat membranes using Latrunculin A. (A and B) Show cells expressing Ede1-mMaple and Sla2-GFP. (C and D) Show cells expressing Pan1-mMaple and Abp1-GFP. The signal from Abp1-GFP was diffuse due to LatA treatment and omitted here. (E and F) Show cells expressing Las17-mMaple and Abp1-GFP. The signal from Abp1-GFP was diffuse due to LatA treatment and omitted here. Boxed regions have been magnified. Scale bars are 1 μm and 100 nm (zoomed regions).

As we relied on formaldehyde fixation to ensure high spatial resolution and localization density in the superresolution images, we next characterized how well fixation preserved endocytic structures. For this, we directly compared the distribution of Ede1, Pan1, and Las17, each from a different functional endocytic module, in living and fixed cells. To achieve optimal resolution in living cells, we treated cells with Latrunculin A (LatA), which arrests endocytosis prior to membrane ingression (Kukulski et al., 2012) and thereby allowed us to image defined, stationary structures. In all cases, the observed structures were very similar between living LatA-treated and fixed LatA-treated cells (Figures 2H and S4). Thus, we concluded that chemical fixation preserved yeast endocytic structures well enough for us to take advantage of the excellent image quality of fixed-cell superresolution imaging.

### Initiating Proteins Form Irregular Structures that Grow over Time

For most endocytic proteins, the individual images resembled their average distribution, hinting toward a fairly constant structural organization during endocytosis. However, the fact that proteins get recruited and disassembled gradually suggests some structural rearrangements over time. Particularly, the early proteins Ede1 and Syp1 that initiate endocytic sites with variable timing on the order of minutes formed highly variable structures, including rings, patches, crescents, lines, and more irregular shapes (Figures S1, S2, and S3), with average outer radii of ∼66 nm (Figures 2C and 2D).

**Figure 3:**
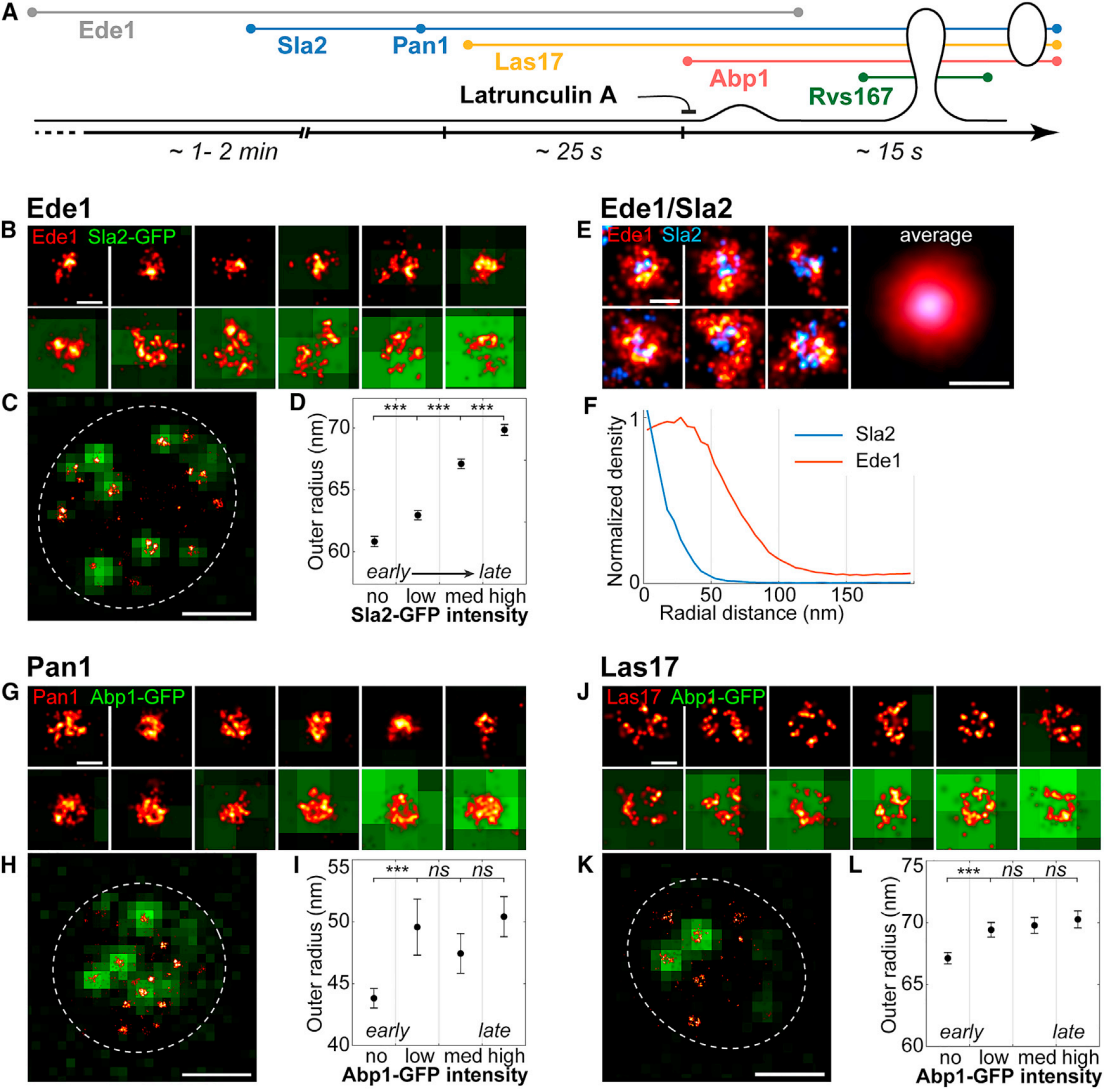
Structural Rearrangements of Key Proteins during Endocytosis (A) Strategy: Staging of endocytosis by combining superresolution imaging with diffraction-limited imaging of Sla2, Abp1, and Rvs167, which each label specific phases of endocytosis. Latrunculin A arrests endocytosis before membrane bending begins (see Figure 2H). (B and C) Ede1 in superresolution overlaid on diffraction-limited images of Sla2-GFP at individual sites (B) and in a cell overview (C). (D) Outer radii of Ede1 sites for no, low, medium, and high Sla2-GFP intensities, as proxy for early to late time points (mean ± SEM; n_no_ = 2514; n_low_ = n_med_ = n_high_ = 2,634). (E) Dual-color superresolution images of Ede1-mMaple and Sla2-GFP αGFP-nanobody-AF647. Representative individual sites and the average distribution from 267 sites are shown. (F) Corresponding radial profiles. (G–I) Like (B)–(D), but for Pan1 in superresolution and Abp1-GFP as timing marker. (n_no_ = 494; n_low_ = n_med_ = n_high_ = 98.) (J–L) Like (B)–(D), but for Las17 in superresolution and Abp1-GFP as timing marker. (n_no_ = 2,550; n_low_ = n_med_ = n_high_ = 1,595.) Scale bars represent 100 nm (B, E, G, and J) and 1 μm (C, H, and K). ^∗∗∗^p < 0.001 from Wilcoxon rank-sum test. See Figure S5 for radial profiles and Table S2 for data.

**Figure S5:**
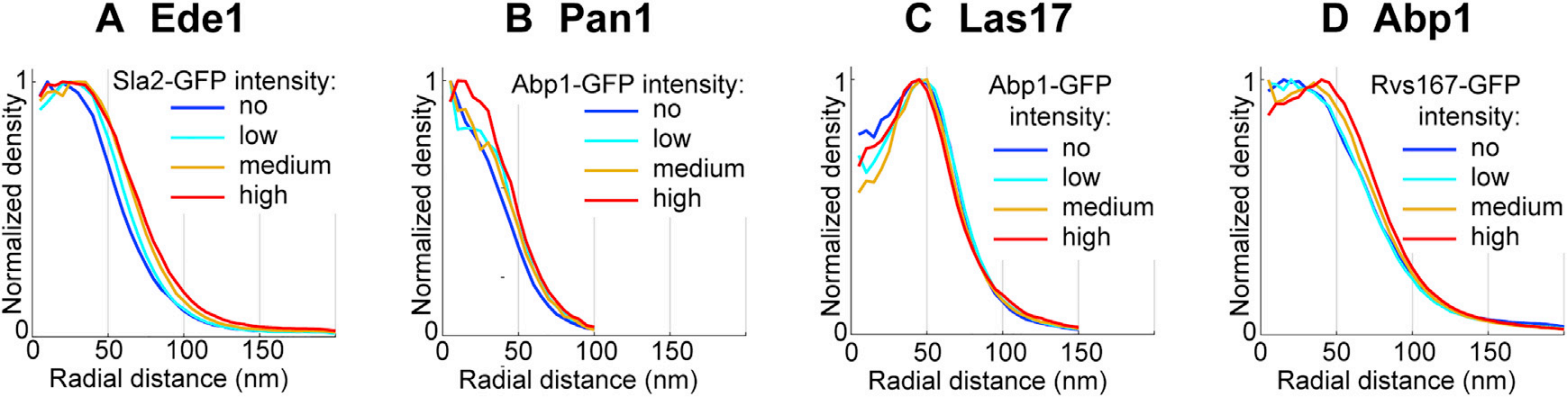
Average Radial Profiles of Ede1, Pan1, Las17, and Abp1 Staged Using Diffraction-Limited Timing Markers, Related to Figures 3 and 5 Average radial profiles of (A) Ede1-mMaple staged by Sla2-GFP, (B) Pan1-mMaple staged by Abp1-GFP, (C) Las17 staged by Abp1-GFP, (D) Abp1 staged by Rvs167-GFP.

To test whether this heterogeneity corresponded to structural changes over time, we combined superresolution imaging of the early protein Ede1 with diffraction-limited imaging of the GFP-tagged coat protein Sla2, whose abundance increases over time until Ede1 is disassembled (Picco et al., 2015), thus providing a reference to sort endocytic sites in time (Figure 3A). We found that the distribution of Ede1 continuously grows in size from an average outer radius of 60.8 ± 0.4 nm (mean ± SEM; n = 2,514; SD = 20.9 nm) up to 69.9 ± 0.4 nm (mean ± SEM; n = 2,634; SD = 22.4 nm) (Figures 3B–3D and S5A).

To further investigate this structural remodeling, we imaged Ede1 and the coat protein Sla2 using dual-color superresolution microscopy. This revealed that Ede1 was arranged either around Sla2 or side by side with it (Figure 3E), but the two proteins rarely overlapped. On average, Ede1 was peripherally organized around Sla2 (Figure 3F).

Together, the growing size of Ede1 structures and the arrangement around Sla2 suggest that the endocytic machinery is initiated with variable structures, which are then remodeled during coat recruitment, potentially by a mechanism where coat proteins form a stable patch occupying the center, and thereby force early proteins to arrange more peripherally.

### Coat Proteins Assemble from Center to Periphery in Patches of Increasing Size

Besides Sla2, a variety of other coat proteins are recruited. These include clathrin (Clc1, Chc1), Ent1, Sla1, Sla2, Pan1, and End3, which on average formed patch-like structures with sizes in a range between 25–50 nm (Figures 2C and 2E). Clathrin, which arrives first, formed the smallest structures, followed by End3, Ent1, and Sla2, and then by the late coat proteins Sla1 and Pan1. Although the averages of all coat proteins were patch-like, part of the individual sites showed ring-like distributions (Figure S1, Figure S2, Figure S3), which agrees with our previous findings that Sla1 can form small rings at endocytic sites (Picco et al., 2015).

We then analyzed how Pan1, which formed the largest structures of the coat, changed over time by imaging Pan1 alongside Abp1-GFP as marker for the actin network (Figures 3G and 3H). Pan1 structures had an average outer radius of 41.8 ± 0.9 nm (mean ± SEM; n = 494; SD = 19.7 nm) before actin polymerization (Figures 3I and S5), which increased to 50.1 ± 1.6 nm (mean ± SEM; n = 98; SD = 16.2 nm) at endocytic sites where actin polymerization had begun, which corresponds to maximum Pan1 recruitment (Kaksonen et al., 2003, Picco et al., 2015). This indicates that Pan1 structures expand during the continuous recruitment to endocytic sites.

Taken together, our data suggest that the progressive assembly of endocytic coat proteins translates into a radially expanding architecture: the early coat proteins clathrin, Sla2 and Ent1, form the core of the coat, whereas the late coat proteins, Sla1 and Pan1, extend to its periphery.

### Nucleation-Promoting Factors and Their Inhibitors Form a Circular Nano-Template for Actin Polymerization

**Figure 4:**
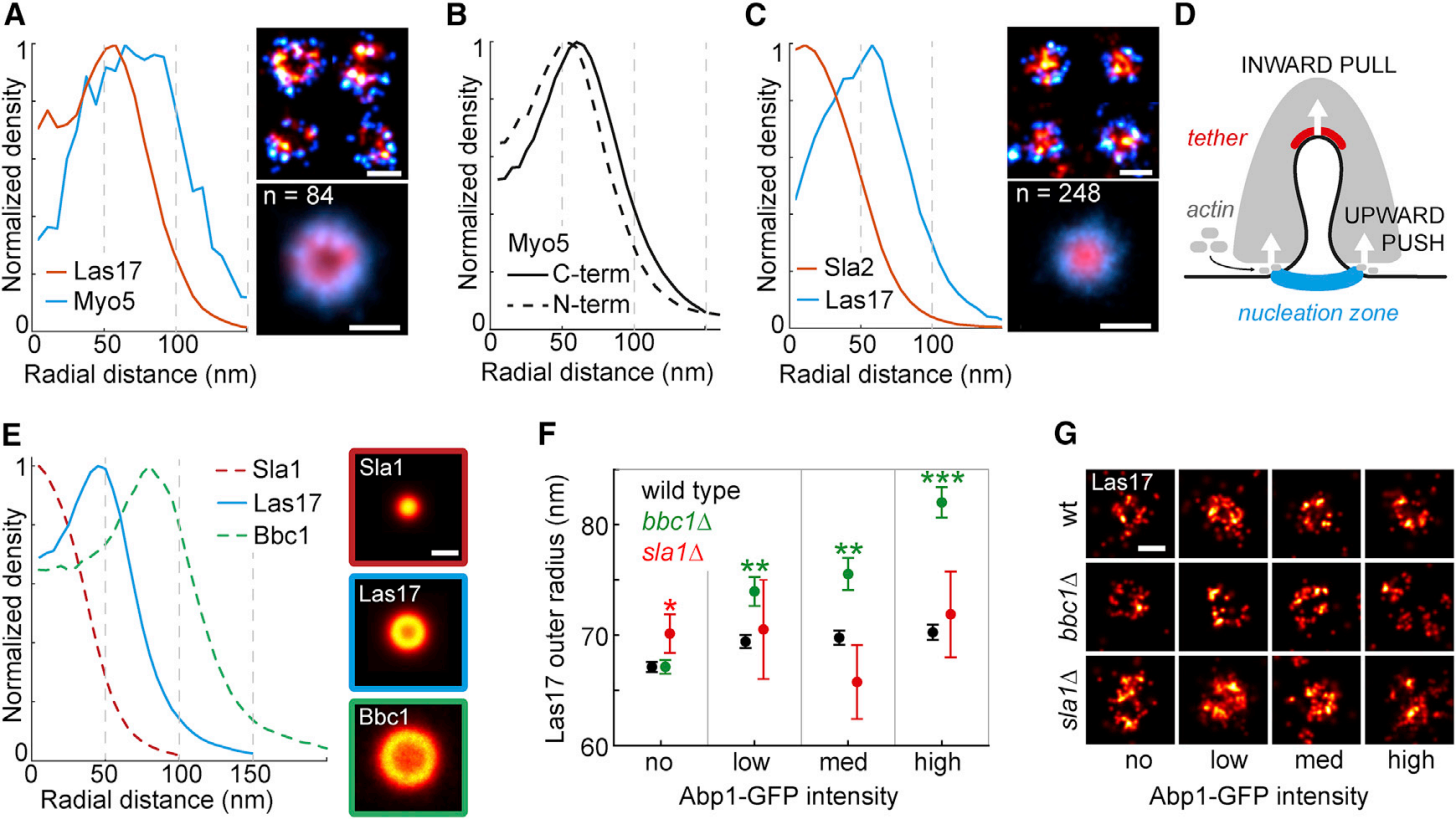
WASP Forms a Nano-Template for Actin Nucleation at the Membrane Base (A) Dual-color superresolution images of Las17-mMaple and Myo5-SNAP at individual endocytic sites, and average. (B) Radial profiles of N-terminally and C-terminally tagged Myo5. (C) Dual-color superresolution images of Las17-SNAP and Sla2-mMaple at individual endocytic sites and the average. (D) Model for force generation by the actin network. (E) Las17 is most abundant between its inhibitors. Shown are average radial profiles and images of Las17, Sla1, and Bbc1. (F) Outer radii of Las17 structures in wild-type (WT), *bbc1Δ*, and *sla1Δ* cells (mean outer radii ± SEM; WT: n_no_ = 2,550, n_low_ = n_med_ = n_high_ = 1,595; *bbc1Δ*: n_no_ = 1,237; n_low_ = n_med_ = n_high_ = 503; *sla1Δ*: n_no_ = 131; n_low_ = n_med_ = n_high_ = 28). ^∗^p < 0.05, ^∗∗^p < 0.01, ^∗∗∗^p < 0.001 from Wilcoxon rank-sum test of WT versus *sla1Δ* (red) and WT versus *bbc1Δ* (green) at each GFP intensity. (G) Individual Las17 sites in WT, *bbc1Δ*, and *sla1Δ* cells for different Abp1-GFP intensities. Scale bars represent 100 nm. See Figure S6 for example images and radial profiles of Las17 *sla1Δ* cells and Table S2 for data.

As the coat is assembling, members of the WASP/Myo module, which regulate actin nucleation start to arrive at endocytic sites, where they formed rings of different sizes visible in their individual images and averages (Figures 2F, S1, S2, and S3). The group of Las17 (yeast WASP), Bzz1, and Vrp1 on average formed rings with indistinguishable radii of ∼70 nm, in agreement with their reported interactions and functional cooperation to promote actin nucleation (Evangelista et al., 2000, Grötsch et al., 2010, Kishimoto et al., 2011, Lewellyn et al., 2015, Sun et al., 2006). By contrast, the rings of the type-I myosins Myo3 and Myo5 were significantly larger with radii of ∼90 nm (Figure 2C). This difference was confirmed in dual-color superresolution images of Myo5 and Las17 at individual endocytic sites, where Myo5 was peripherally arranged around a Las17 ring (Figure 4A).

The discrepancy of ∼20 nm between Vrp1 and Myo5 radii was surprising, as Vrp1 is required to recruit Myo3/5 to endocytic sites (Grötsch et al., 2010, Lewellyn et al., 2015). Thus, we hypothesized that Myo5 could assume an extended conformation. Indeed, when we endogenously tagged Myo5 with mMaple at its N terminus, we found its N terminus to be on average ∼14 nm further inside than the C terminus (Figure 4B). This indicates a preferential radial orientation of Myo5, where its motor domain points inward toward the center of the endocytic site. Whether this arrangement facilitates an interaction with the more centrally located Vrp1—and what it implies for myosins’ mode of action during endocytosis—should be the subject of future studies.

The WASP protein Las17 arrives at endocytic sites together with the most peripheral coat protein Pan1 around 20 s before actin polymerization begins. Las17 is therefore the first protein to form clear ring-like structures. Thus, we asked whether Las17 is pre-patterned as a ring already on the flat plasma membrane or whether its ring-like distribution is caused by the invaginating membrane. When we arrested endocytosis at a flat membrane using LatA (Kukulski et al., 2012), Las17 still formed rings with a slightly larger outer radius of 74.2 ± 0.7 nm (mean ± SEM; n = 2,179; SD = 31.6 nm) (Figure 2H), demonstrating that ring structures of nucleation promoting factors can form on the flat membrane in the absence of polymerized actin. Next, we analyzed whether Las17 rings changed in size during membrane ingression by imaging Las17 alongside Abp1-GFP as a temporal reference for actin polymerization (Figures 3J and 3K). We found that Las17 clearly formed rings prior to membrane bending that barely increased in size from 67.1 ± 0.5 nm (mean ± SEM; n = 2,550; SD = 23.1 nm) to 70.2 ± 0.7 nm (mean ± SEM; n = 1,595; SD = 27.4 nm) (Figures 3L and S5C) during actin polymerization. We conclude that Las17 forms robust ring structures with a radius of ∼70 nm early on the flat plasma membrane before actin polymerization begins.

Why then does Las17 form a ring, when the previously assembled coat proteins form patches and actin polymerization has not started to invaginate the membrane? The core coat proteins clathrin, Sla2 and Ent1 form a tight molecular lattice *in vitro* (Skruzny et al., 2015), and might prevent later arriving proteins from accessing the center of the endocytic site, thus determining the minimal size of the ring they form. Consistent with this hypothesis, dual-color superresolution imaging of Las17 and the coat protein Sla2 showed that Sla2 was located inside the Las17 rings (Figure 4C). Taken together, our data revealed that actin nucleation is pre-patterned, already on a flat membrane, by a ring of Las17 molecules around the endocytic coat in its center. Actin monomers are therefore added to filaments close to this nucleation zone. This induces an upward push of the actin network, which is attached to the membrane in the center by Sla2 and Ent1/2 molecules, explaining how polymerizing actin can pull the plasma membrane inward (Figure 4D).

Interestingly, coat proteins may not only set the physical inner limit of actin nucleation by Las17, but may also ensure this boundary biochemically, as the peripheral coat protein Sla1 is a WASP inhibitor (Rodal et al., 2003). Thus, we asked whether there is also an outer limit of actin nucleation. Bbc1, another WASP inhibitor (Rodal et al., 2003), formed the largest rings of the WASP/Myo module with a radius of ∼98 nm. This arrangement could indicate that the ring of Las17 is regulated on its inside by a patch of Sla1 and its outside by a ring of Bbc1, thereby controlling Las17 activity for actin nucleation in its radial zone at ∼70 nm (Figure 4E).

To test whether this distinct organization of Las17 and its inhibitors is functionally important, we perturbed it by deleting *BBC1*. We then analyzed the distribution of Las17 over time using Abp1-GFP as timing marker for actin polymerization. In cells lacking Bbc1, Las17 initially formed the same sized rings like in wild-type cells. However, once actin polymerization began, Las17 significantly expanded from the outer radii of 67.1 ± 0.6 (mean ± SEM; n = 1,237; SD = 21.6 nm) to 82.0 ± 1.4 nm (mean ± SEM; n = 503; SD = 30.8 nm) and became more irregular and fragmented (Figures 4F and 4G). Thus, upon actin polymerization, Bbc1 does not merely act as biochemical inhibitor of Las17, but rather regulates the localization of Las17.

**Figure S6:**
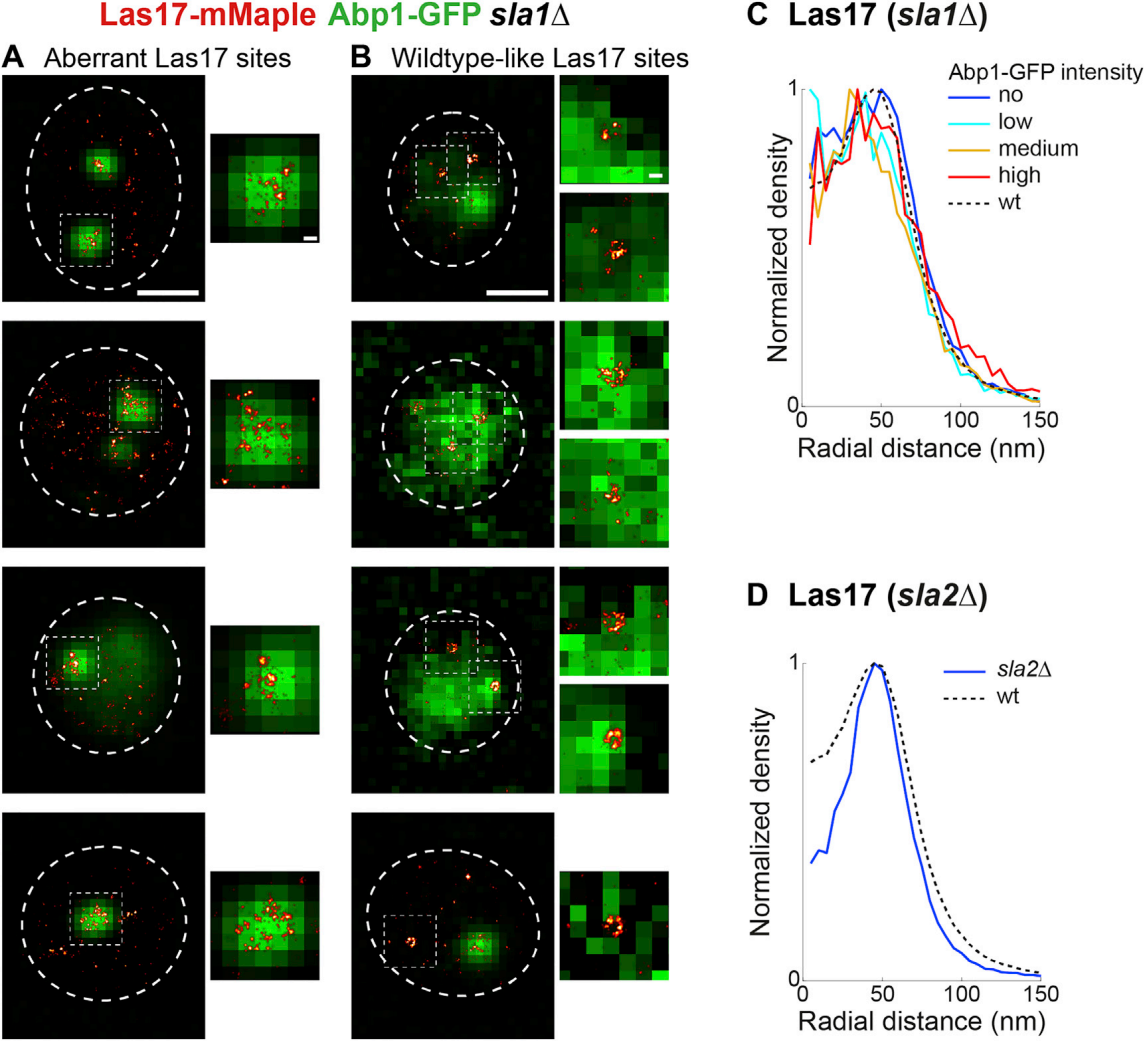
Las17 Structures in *sla1Δ* Cells, Related to Figure 4 (A and B) *SLA1* deletion leads to strong changes in Las17 structures. Shown are example *sla1Δ* cells expressing Las17-mMaple and Abp1-GFP. We note that the large majority of cells showed Las17 in large cluster-like structures (A), while a small subset contained Las17 structures that were similar in size to wild-type (B). (C) Shows radial profiles of Las17 in *sla1Δ* cells for different Abp1-GFP intensities (compare Figures 3J–3L). (D) Shows radial profile of Las17 in *sla2Δ* cells. Scale bars 1 μm or 100 nm (zoomed regions).

We also imaged Las17 in *sla1Δ* cells, which have a much stronger phenotype than *bbc1Δ* cells, (Ayscough et al., 1999, Howard et al., 2002, Kaksonen et al., 2005, Sun et al., 2015). There, Las17 almost always formed large irregular clusters associated with large actin patches (Figure S6A). At the remaining small subset of sites (215 sites out of ∼5,000 cells), Las17 was distributed similar to wild-type (Figures 4F, S6B, and S6C). Likewise, Las17 formed rings in *sla2Δ* cells (Figure S6D), suggesting that no single coat protein alone defines the inner boundary of Las17 rings.

Taken together, we have discovered that the major WASP family actin nucleation promoting factor Las17 forms a precisely controlled template for actin nucleation from a ring of ∼70 nm in radius. During actin polymerization, Bbc1 is important to maintain Las17 ring size. This molecular architecture is established on the flat plasma membrane before actin polymerization, and enables the growing actin network to exert a pulling force and create an invagination.

### Temporal Reconstruction Shows Dynamic Nucleation of Actin from the WASP Nucleation Zone

**Figure 5:**
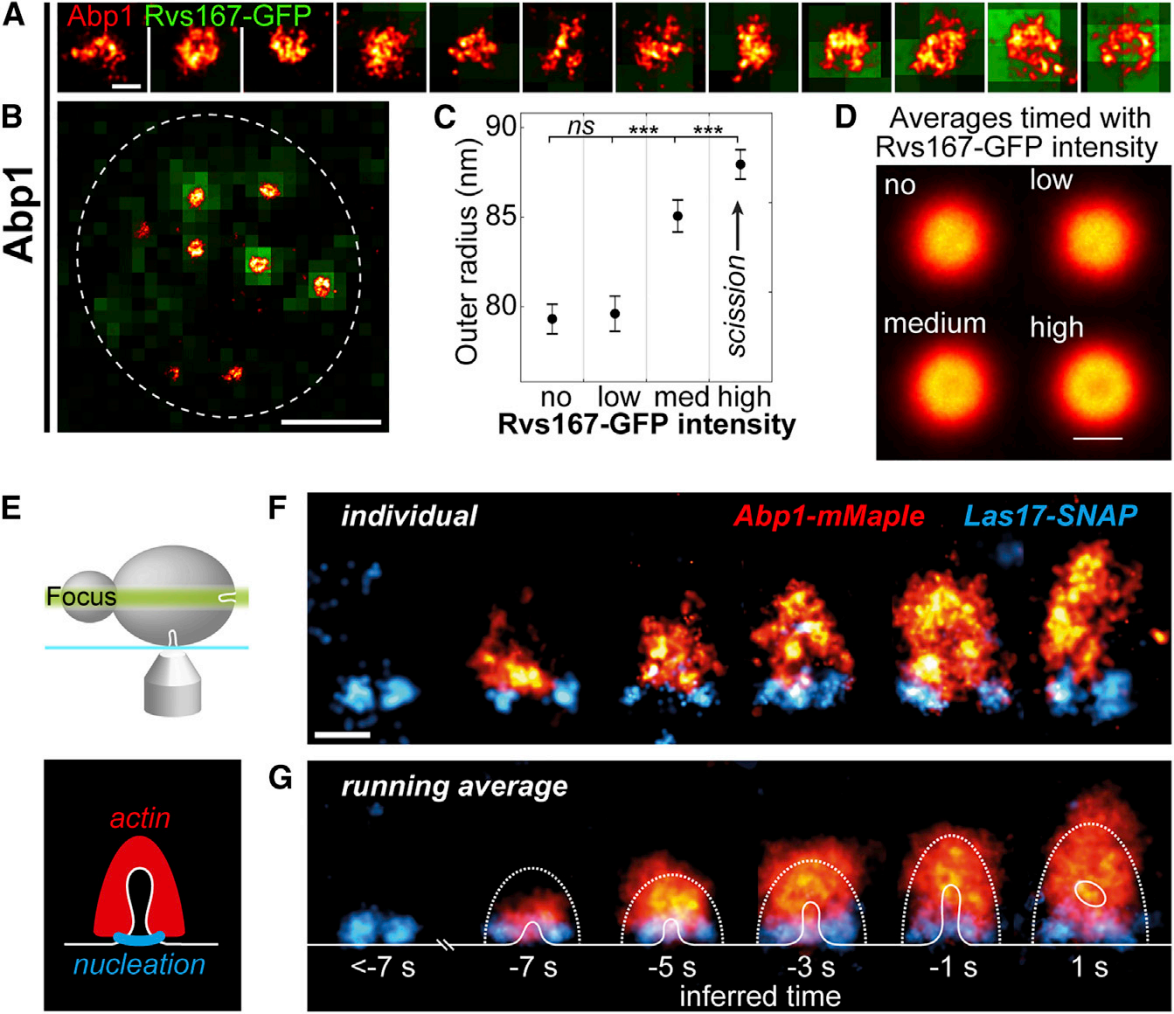
The Actin Network Emanates from the WASP Nucleation Zone (A and B) Abp1 in superresolution overlaid with diffraction-limited Rvs167-GFP as timing marker for vesicle scission at individual sites (A) and in a cell overview (B). (C) Outer radius of Abp1 for no, low, medium, and high Rvs167-GFP intensities (mean ± SEM; n_no_ = 1,044; n_low_ = n_med_ = n_high_ = 568; data in Table S2). (D) Average images of Abp1 for each time window. At medium and high Rvs167-GFP, a pronounced minimum in the center indicates the membrane invagination. See Figure S5 for radial profiles. (E) Schematic of the side-view perspective used in (F) and (G). (F) Dual-color side-view superresolution images of Las17-SNAP and Abp1-mMaple at individual sites. Images were rotated so endocytosis occurs upward, and sorted by the distance of Abp1 centroid to Las17 at the base. (G) Running-window averages of Las17 and Abp1 at endocytic sites. For comparison, average outer boundaries of the actin network (dotted lines), and average plasma membrane profiles (solid line) obtained by CLEM (Kukulski et al., 2012) are overlaid for each time point, as inferred from the images. ^∗∗∗^p < 0.001 from Wilcoxon rank-sum test. Scales bars represent 100 nm (A, D, and F) and 1 μm (B). See also Figure S5 and Table S2.

**Figure S7:**
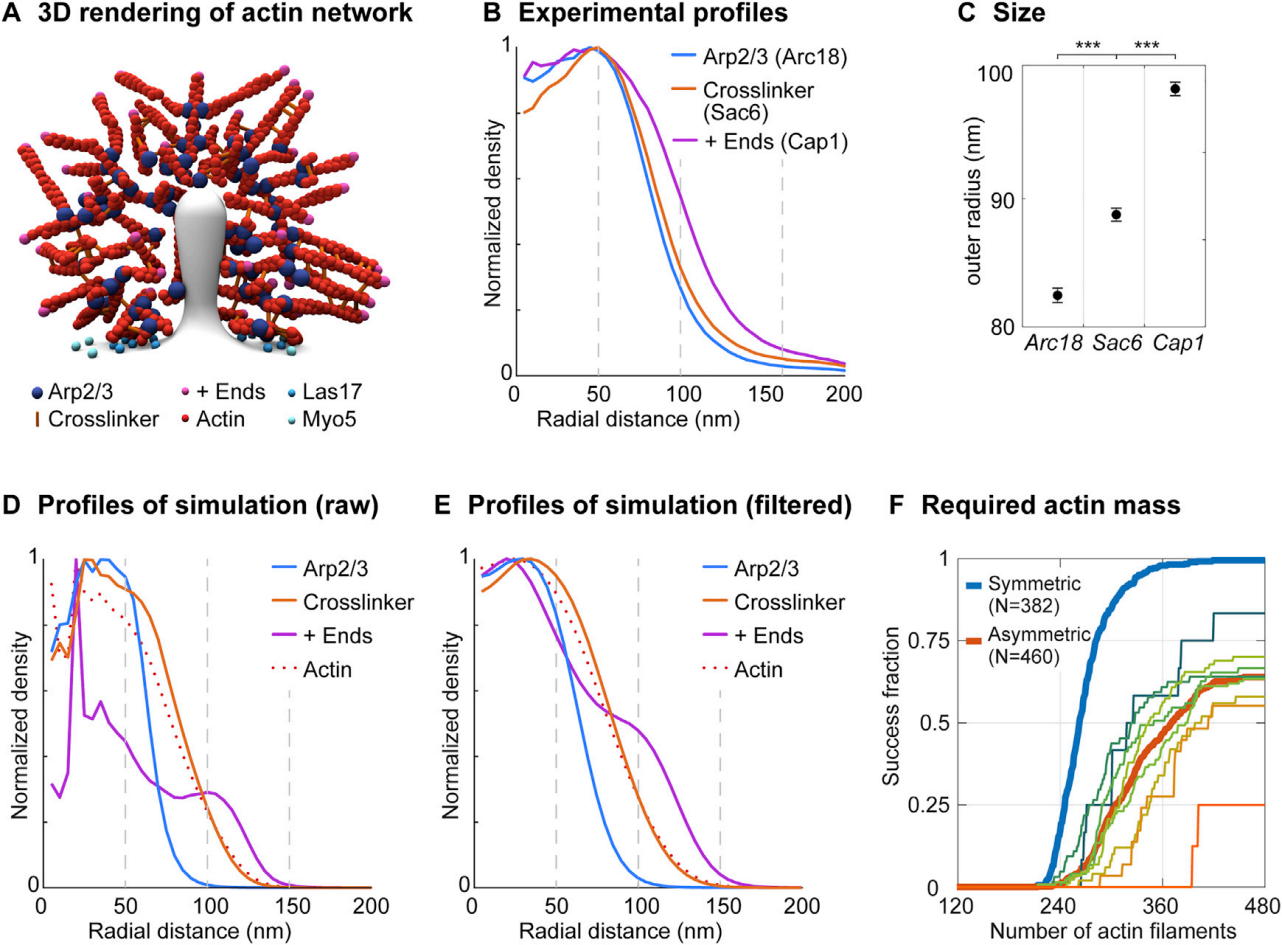
Potential Organization of Actin Filaments, Related to Figure 6 (A) Illustration of a thin slice through an endocytic actin network. (B and C) The experimental radial profiles (B) and mean outer radii ± SEM of Cap1, Arc18 and Sac6 (C). These are indicating that barbed ends (Cap1) protrude further outward than pointed ends (Arc18). Crosslinker (Sac6) had an intermediate size, consistent with a cross-linking function between the middles of the filaments (Skau et al., 2011). (D and E) This organization is recapitulated in the raw (D) and filtered (E) average profiles from Cytosim simulations. Profiles in (E) were calculated from (D) by blurring the raw coordinates from the simulations by 15 nm to simulate the localization precision of superresolution imaging. (F) Comparison how many actin filaments are needed to reach a certain invagination depth in symmetric and asymmetric nucleation. ^∗∗∗^p < 0.001 from Wilcoxon rank-sum test.

During the late phase of endocytosis, the plasma membrane is invaginated by polymerizing actin. Here, we determined the average radial distribution of the actin network components Abp1, Arc18, Cap1, Cap2, Crn1, Sac6, and Twf1, all of which formed similar large structures corresponding to the big endocytic actin network (Figures 2A–2C and 2G). Using the amphiphysin Rvs167-GFP as timing marker, we then showed that Abp1 structures were biggest (outer radius 87.9 ± 0.8 nm; mean ± SEM; n = 568; SD = 19.7 nm) and had a pronounced minimum in their center at the time point of vesicle scission (Figures 5A–5D and S5D). This matches the expected distribution resulting from a dome-like actin network encompassing the invagination (Idrissi et al., 2008, Kukulski et al., 2012, Mulholland et al., 1994). Moreover, the size differences of the other actin module proteins revealed that barbed ends of filaments are on average oriented outward further than the pointed ends (Figure S7), agreeing with a previous model (Berro and Pollard, 2014b).

Next, we directly visualized the Las17 nucleation zone and the resultant actin network using dual-color superresolution microscopy of Las17 and Abp1 in a side-view configuration (Figure 5E). Whereas Las17 always localized close to the base of the plasma membrane, Abp1 formed structures of different shapes and sizes corresponding to different stages of actin network formation. To infer the endocytic time of our snapshots, we used previously reported live-cell findings that the center of mass of Abp1 is continuously moving inward during endocytosis (Picco et al., 2015). Thus, we ordered our snapshots in time by sorting them by increasing distance between the Abp1 centroid and Las17 at the base. The resultant temporal reconstruction revealed that the actin network emanates directly above Las17 and progressively grows into the cytoplasm (Figures 5F and 5G). The average structures were ∼200 nm wide in all but the first time point and increased in height from ∼70 nm to ∼240 nm. This agrees well with previous measurements using CLEM on high-pressure frozen cells (Figure 5G) (Kukulski et al., 2012), thus validating our approach and confirming good structural preservation in our chemically fixed samples.

In summary, ordering our snapshot data in time directly shows how the Las17 nano-template guides actin polymerization.

### Brownian Dynamics Simulations Show that Symmetric Actin Polymerization around the Invagination Increases the Efficiency of Endocytosis

During endocytosis, the turgor pressure opposes membrane invagination with forces exceeding 1000 pN (Basu et al., 2014, Dmitrieff and Nédélec, 2015), which is much higher than the polymerization stall force of single actin filaments of 1–10 pN (Footer et al., 2007, Kovar and Pollard, 2004). Nevertheless, endocytic vesicles are formed with high efficiency and uniform size (Kaksonen et al., 2003, Kukulski et al., 2012). We thus speculated that nanoscale patterning of actin nucleation could be important for the actin network to generate the force required to form vesicles. To test this, we simulated the mechanics of the dynamic endocytic actin network using the open-source modeling framework Cytosim (Nédélec and Foethke, 2007).

**Figure 6:**
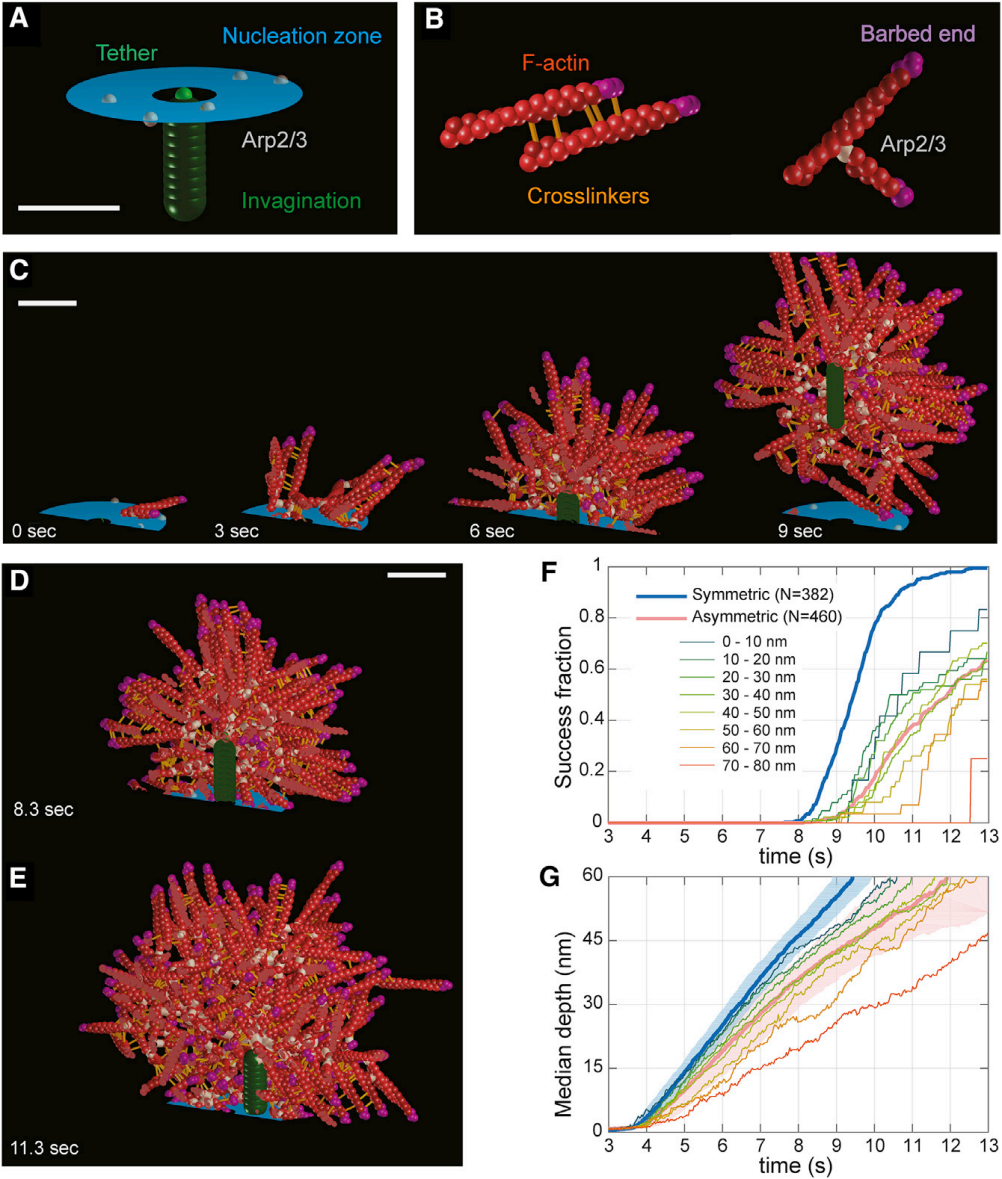
Simulations of the Actin Network at Endocytic Sites Using Cytosim (A and B) Initial configuration of the simulation (A) and overview of simulated elements (B). (C) Time series of a representative simulated endocytic event, with a snap-through transition at ∼9 s. (D) Detail of an endocytic event where nucleation occurred all around the invagination. 5,022 actin monomers were required to reach the snap-through transition. Lateral distance between actin center of mass and invagination is 7 nm. See also Video S1. (E) Detail of an endocytic event where actin nucleation occurred asymmetrically with respect to the invagination, shown at the same depth as (D). More time and actin (7,824 monomers) were required to reach the snap-through transition. Actin is not symmetrically organized, with a lateral distance of 32 nm between actin center of mass and invagination. (F) Fraction of successful simulations that overcome the snap-through transition over time, for symmetric (blue) and asymmetric nucleation (red). N is number of simulations. Thin lines correspond to different distances between tether and center of actin nucleation (bin width, 10 nm) at the starting time of invagination. (G) Median invagination depth over time for symmetric (blue) and asymmetric nucleation (red). Color-filled areas represent the spread from the first to third quartile. Thin lines show median invagination depths over time within the same bins as (F). Scale bars represent 50 nm. See also Figure S7 and Videos S2 and S3.

We modeled the actin network with a minimal set of components and experimentally measured parameters. Based on our findings, we implemented a ring-shaped nucleation zone (Figure 6A) where activated Arp2/3 complex continuously appears and creates new filaments from existing ones at a 70° angle (Figure 6B) (Mullins et al., 1998). A single filament acts as first nucleation site, consistent with a “sever, diffuse, and trigger” model where short filaments from fragmenting actin patches activate Arp2/3 complex at new endocytic sites (Chen and Pollard, 2013). Filaments grow with Brownian ratchet dynamics and a stall force of 9 pN (Dmitrieff and Nédélec, 2016) up to a length of 60 nm, which is in between estimates of filament lengths at endocytic sites in budding and fission yeast (Berro et al., 2010, Picco et al., 2015). Crosslinkers are modeled as 10 nm-long elastic linkers with a 10 pN unbinding force (Miyata et al., 1996) (Figure 6B). Actin is strongly bound to the tip of the invagination, mimicking the function of Sla2 and Ent1/2 (Kaksonen et al., 2003, Skruzny et al., 2012) (Figure 6A). The required force is 200 pN at first and then increases up to 1,000 pN at a depth of 60 nm. There, the invagination reaches a previously proposed “snap-through” transition (Dmitrieff and Nédélec, 2015, Walani et al., 2015), and endocytosis is regarded as successful.

In these simulations, actin filaments formed a branched network that reliably produced forces beyond 1000 pN and thus overcame turgor pressure (Figure 6C; Video S1). Around 60 actin filaments were necessary to complete endocytosis, similar to previous reports (Picco et al., 2015), and the average filament orientation agreed between simulations and superresolution data, where barbed ends were further outside than the pointed ends bound by Arp2/3 and crosslinkers were in between (Figure S7).

Video S1. Cytosim Simulation of Symmetric Actin Nucleation Shows Successful Endocytosis, Related to Figure 6 Different views of a simulated symmetric endocytic event. Actin filaments are shown in red, barbed ends in purple. Arp2/3 complex (light gray) appears in the nucleation zone (blue) at the cell wall (not shown). The membrane invagination (dark green) is constrained to the center of the nucleation zone. The top-left view is a cut-through view, only showing the back half of the structure. In the bottom-left view the structure is seen from the top (i.e., from the center of the cell). In the two right views, actin is hidden, to better see the invagination from the side (top) and the top (bottom).

Video S2. Cytosim Simulation of Asymmetric Actin Nucleation Shows Slow Endocytosis, Related to Figure 6 Different views of a simulation where actin nucleation occurred asymmetrically with respect to the invagination, which was simulated by not restricting the invagination to the center of the nucleation zone. Colors as in Video S1. Top-left: actin machinery as a cut-through view, only showing the back half of the structure. In the bottom-left view the structure is seen from the top (i.e., from the center of the cell). In the two right views, actin is hidden, to better see the invagination from the side (top) and the top (bottom).

Video S3. Cytosim Simulation of Asymmetric Actin Nucleation Shows Unsuccessful Endocytosis, Related to Figure 6 Colors and views as in Videos S1 and S2. Leftmost 4 panels show a simulation where actin nucleation was asymmetric, and endocytosis was unsuccessful. For comparison, the rightmost 4 panels show a simulated endocytic event with symmetric nucleation.

With this computational model, we next tested whether a ring-shaped nucleation zone is advantageous for the efficiency of vesicle budding. A key advantage of a ring-shaped nucleation zone could be that membrane invagination occurs in its center, with actin polymerizing symmetrically around it, preventing lateral displacement or tilting of the invagination.

To test this, we simulated endocytosis with two different geometries, either where actin nucleation occurred symmetrically all around the invagination (Figure 6D, Video S1) or where filaments could be nucleated asymmetrically with respect to the invagination, which we simulated by not constraining the invagination to the center of the nucleation zone (Figure 6E, Video S2).

When we calculated endocytic success rates, we indeed found that endocytic events were far more successful when actin nucleation occurred all around the central invagination (Figure 6F), consistent with the arrangement of Las17 around Sla2 that we observed in our images (Figures 4C and 4D). Moreover, the further actin nucleation activity and invagination were apart, the less likely endocytosis was to succeed (Figure 6F) and the slower it proceeded (Figure 6G, Video S3). These results indicate that actin-driven pulling on the membrane is much more efficient in creating invaginations when they are formed in the center. When actin nucleation was asymmetric, invaginations often moved sideways in our simulations, because the actin network exerted a lateral force on the invagination, rather than pulling it inward. Thus, more time (Figure 6G) and more actin filaments (Figure S7F) were required to reach any invagination depth than when actin nucleation was symmetric around the invagination.

We conclude that the ring-like actin nucleation nano-template ensures high efficiency of vesicle budding by confining the membrane invagination at its center, triggering actin polymerization all around it and thereby creating a force perpendicular to the plasma membrane.

## Discussion

### The Endocytic Machinery Is Radially Assembled on the Flat Membrane

Here, we showed that proteins are organized in distinct nanoscale radial zones that correlate with function. The early proteins Ede1 and Syp1 were distributed similarly, which is expected as they directly interact and Syp1 recruitment requires Ede1 (Boeke et al., 2014, Stimpson et al., 2009). The variable structures of Ede1 are remodeled and become preferentially organized around the growing coat (Figures 3B–3F), potentially because Ede1 competes with late coat proteins like Pan1 for binding to the early coat proteins Ent1/2 via their EH domains. We propose that the structural remodeling of early proteins upon coat formation represents a structural switch from the variable initiation phase into the late phase of endocytosis that leads to vesicle budding with highly stereotypic timing.

In the central coat, Sla2 and Ent1/2 bind the membrane and clathrin, forming a lattice (Skruzny et al., 2015), while Pan1 and Sla1 extend the patch in size, consistent with the recently reported organization of their homolog Intersectin within the endocytic coat in mammalian cells (Sochacki et al., 2017). The coat is likely stable, as its components exchange very slowly at least in LatA treated cells (Skruzny et al., 2012).

Subsequently, WASP/Myo module proteins are recruited in distinct ring-shaped zones. Arrival time and size are again correlated: Las17, Vrp1, and Bzz1 all directly interact (Sun et al., 2006) and form rings of similar size around the coat. When actin polymerization begins, Myo3 and Myo5 are recruited and form larger rings, and Bbc1 forms the largest rings around the coat.

**Figure 7:**
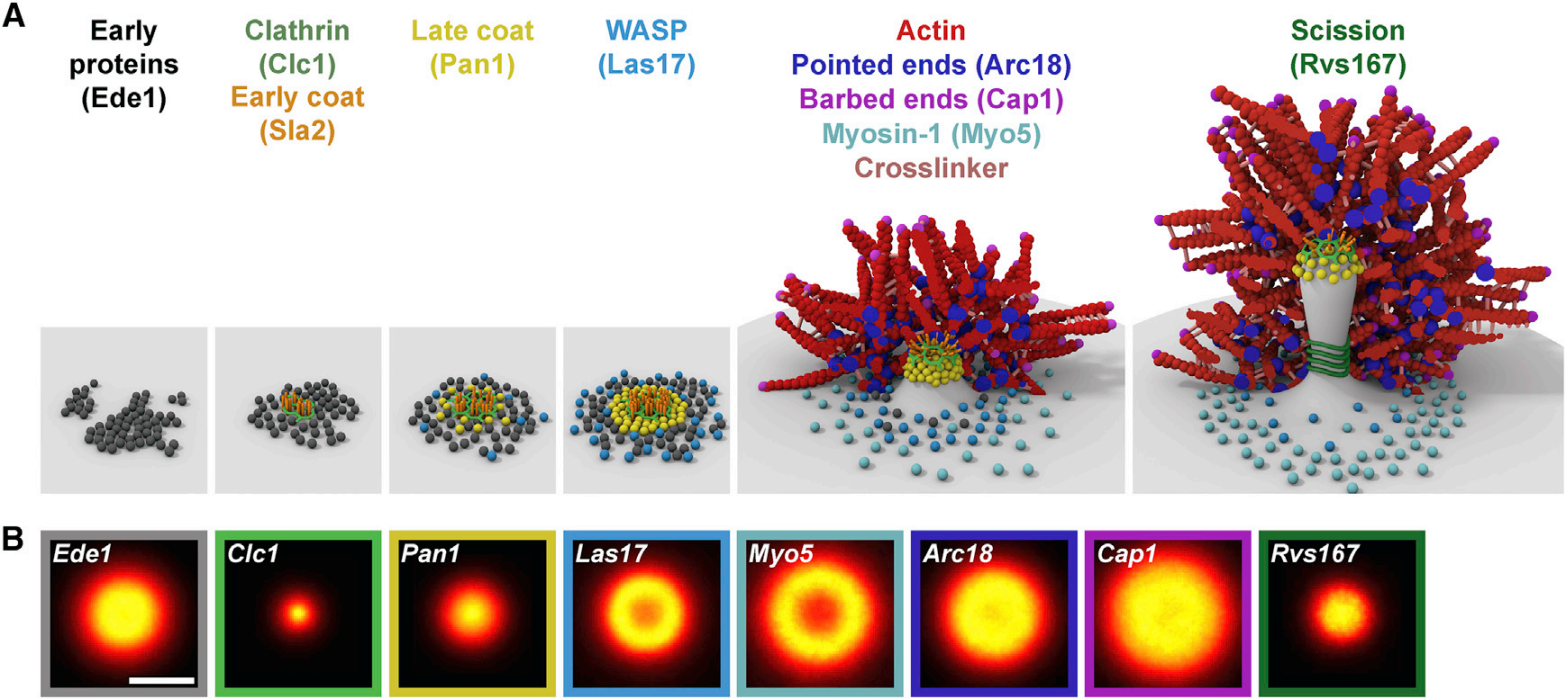
The Endocytic Machinery Assembles via Peripheral Binding (A) Schematic representation of the assembly of the endocytic machinery (for description, see text). (B) Representative radial averages of endocytic proteins at key time points during assembly. Shown are Ede1 (early), Clathrin (Clc1, early coat), Pan1 (late coat), Las17 (WASP), Myo5 (Myosin-1), Arc18 (Arp2/3 complex bound to pointed ends), Cap1 (barbed ends), and Rvs167 (Scission). Scale bar represents 100 nm.

Taken together, our results reveal different stages by which the endocytic machinery is collectively assembled on a flat plasma membrane (Figure 7). After initiation, the coat forms, and newly arriving proteins are recruited to its periphery into distinct radial zones, which are determined by a variety of interactions with proteins that are already present at the endocytic site.

### A Nano-Pattern of Actin Nucleation Explains Efficient and Robust Membrane Invagination

In yeast, actin polymerization provides the necessary force to bend the plasma membrane, which is much higher than in mammalian cells because yeast have a turgor pressure (Aghamohammadzadeh and Ayscough, 2009). We found that actin nucleation, which is rate-limiting for actin polymerization (Pollard and Cooper, 2009), is patterned by a ring of Las17 around the coat at the plasma membrane, and visualized how an actin network is formed from this nucleation nano-template. Using simulations of actin polymerization at endocytic sites, we could show that this ring-shaped nucleation zone is crucial for fast and efficient vesicle formation.

In light of the inherent stochasticity of macromolecular machineries, the robustness and efficiency of endocytosis in yeast is remarkable. Endocytosis is virtually always productive (Kaksonen et al., 2005) and occurs with high spatio-temporal regularity (Berro and Pollard, 2014a, Picco et al., 2015). Interestingly, to pull in the membrane, high forces are already required shortly after membrane ingression begins, when the invaginations are still shallow (Dmitrieff and Nédélec, 2015, Hassinger et al., 2017, Walani et al., 2015). Pre-patterning actin nucleation around the center, where membrane and actin are physically linked by Sla2 and Ent1/2 (Figures 4C and 4D), allows rapid filament nucleation from the start, centers the membrane tethers, lends directional stability to the actin machinery, and thereby ensures the endocytic machinery produces enough force during its most crucial phase at the beginning of invagination.

The mechanistic relevance of this nucleation zone is highlighted in *bbc1Δ* cells, where Las17 initially formed rings like in wild-type cells, but with the onset of actin polymerization became larger and less regular. It was previously shown that in *bbc1Δ* cells, the coat protein Sla1 moves in further than in wild-type cells and that more actin is assembled, resulting in a wider actin network (Kaksonen et al., 2005, Picco et al., 2018). A larger Las17 nucleation zone explains this phenotype, where a bigger, wider actin network can propel the forming vesicle further into the cytoplasm.

Moreover, it was recently shown that Las17 at endocytic sites is activated when a threshold concentration is reached (Sun et al., 2017), indicating that precise control of its nanoscale localization is important to maintain the correct regulation of Las17.

### Role of Type I Myosins at Endocytic Sites

The type I myosins Myo3/5 were arranged in rings around Las17 (Figure 4A), with their N-terminal motors preferentially oriented inward (Figure 4B). Interestingly, an opposite radial orientation of Myo3/5 has been hypothesized previously, where N-terminal motor domains were proposed to capture peripheral actin filaments, and tether them to the plasma membrane (Lewellyn et al., 2015). The arrangement found here rather suggests a mode of action where motors bind to actin filaments closer to the center, and possibly exert a power stroke to translocate filaments to assist endocytosis (Kaksonen et al., 2006, Lewellyn et al., 2015).

Our findings of a ring-shaped Las17 nucleation zone with Myo3/5 around it are particularly interesting in light of a recent study that visualized several components of endocytic actin patches in fission yeast (*S. pombe)* using live-cell superresolution microscopy (Arasada et al., 2018). Using a side-view perspective (equivalent to Figure 5E), the study reported that Wsp1p and Myo5p (*S. pombe* homologs of Las17 and Myo3/5) first localized to the membrane base. Wsp1p then moved inward, while Myo1p remained at the membrane, creating two distinct actin networks which push against each other to mediate vesicle scission.

Here, we have found that in *S. cerevisiae* Las17 and Myo3/5 are spatially separated in radial zones at the base of the membrane, and we anticipate this organization is present in *S. pombe* as well. In contrast to Wsp1p in *S. pombe,* we have shown that in *S. cerevisiae* Las17 remains close to the base of the membrane (Figures 5F and 5G), in line with previous reports using fluorescence microscopy (Kaksonen et al., 2005, Picco et al., 2015, Sun et al., 2006, Sun et al., 2017). Whether the functional consequences of the spatial organization of Las17 and Myo5 are related to the proposed mechanism of *S. pombe* Wsp1p and Myo1p remains unknown.

### Possible Role of WASP Nucleation Zones in Animal Cells

The machinery of clathrin-mediated endocytosis is highly conserved from yeast to humans (Weinberg and Drubin, 2012), with actin, Arp2/3 complex, WASP, and other actin regulators being present, but not at all sites and in variable amounts (Li et al., 2015, Taylor et al., 2011). Like in yeast, actin is essential when high forces are needed for membrane deformation, examples of which involve endocytosis at cellular adhesions (Batchelder and Yarar, 2010, Liu et al., 2009), of large cargo particles (Cureton et al., 2009), and when membrane tension is high (Boulant et al., 2011). In these cases, branched actin networks are formed at the base of clathrin-coated pits (Collins et al., 2011), which indicates that actin nucleation by the Arp2/3 complex could be patterned there by WASP similar to what we have shown here.

Actin polymerization is essential for membrane remodeling in various other cellular processes. During cell migration, WASP activates Arp2/3-mediated filament nucleation at the leading edge to push forward the membrane (Pollard and Cooper, 2009). Recently, WASP was shown to act as distributive polymerase on the membrane, where it dramatically accelerates growth of actin filaments (Bieling et al., 2018). A nano-template of concentrated WASP molecules might thus assist nearby filaments to grow faster than surrounding ones, which for instance is a requirement for filopodia formation in dynamic lamellipodia (Bieling et al., 2018).

In summary, the data presented in this work substantially improve our understanding how the complex and dynamic endocytic machinery produces vesicles with high precision and efficiency. We believe our data will be useful for further mechanistic modeling of the endocytic machinery and anticipate that the combination of our and other complementary approaches, including live-cell fluorescence and superresolution imaging and EM, will continue to be fruitful for future studies of endocytosis.

## STAR★Methods

### Key Resources Table

**Table:** 

REAGENT or RESOURCE	SOURCE	IDENTIFIER
**Chemicals, Peptides, and Recombinant Proteins**		

4-Aminobenzoic acid	Merck	Cat#822312; CAS: 150-13-0
5-Fluoroorotic Acid Monohydrate	Toronto Research Chemicals	Cat#F595000; CAS: 220141-70-8
Adenine	Sigma-Aldrich	Cat#A8626; CAS: 73-24-5
Adenine hemisulfate salt	Sigma-Aldrich	Cat#A3159; CAS: 321-30-2
Ammonium Chloride	Merck	Cat#101145; CAS: 12125-02-9
anti-GFP nanobody conjugated to Alexa Fluor 647	Custom made	N/A
Bacto Agar	BD Biocsciences	Cat#214010
Bovine Serum Albumin	Sigma-Aldrich	Cat#A7030; CAS: 9048-46-8
Catalase from bovine liver	Sigma-Aldrich	Cat#C3155; CAS: 9001-05-2
Concanavalin A	Sigma-Aldrich	Cat#C2010; CAS: 11028-71-0
Cysteamine	Sigma-Aldrich	Cat#30070; CAS: 60-23-1
D-Galactose	Serva	Cat#22020; CAS: 59-23-4
D-Sorbitol	Sigma-Aldrich	Cat#S3889; CAS: 50-70-4
D(+)-Glucose monohydrate	Merck	Cat#104074; CAS: 14431-43-7
Deuterium Oxide (99.8%)	euriso-top	Cat#D216L-MPT; CAS: 7789-20-0
DMSO, anhydrous	Sigma-Aldrich	Cat#276855; CAS: 67-68-5
dNTP Mix	ThermoFisher Scientific	Cat#R0191
DTT	Sigma-Aldrich	Cat#43819; CAS: 3483-12-3
EDTA	Merck	Cat#108418: CAS: 6381-92-6
FastAP Thermosensitive Alkaline Phosphatase	ThermoFisher Scientific	Cat#EF0651
FastDigest BamHI	ThermoFisher Scientific	Cat#FD0054
FastDigest *Nco*I	ThermoFisher Scientific	Cat#FD0573
FastDigest SalI	ThermoFisher Scientific	Cat#FD0644
Formaldehyde solution about 37%	Merck	Cat#104003
Glucose Oxidase from *Aspergillus niger*	Sigma-Aldrich	Cat#49180; CAS: 9001-37-0
Glutamine	Sigma-Aldrich	Cat#P0380; CAS: 147-85-3
Glycine	Sigma-Aldrich	Cat#G7126; CAS: 56-40-6
Hydrochloric acid fuming 37%	Merck	Cat#100317
Hygromycin B	Roth	Cat#CP13; CAS: 31282-04-9
Image-iT FX Signal Enhancer	ThermoFisher Scientific	Cat#I36933
L-Alanine	Sigma-Aldrich	Cat#A7627; CAS: 56-41-7
L-Arginine	Sigma-Aldrich	Cat#A5006; CAS: 74-79-3
L-Asparagine	Sigma-Aldrich	Cat#A0884; CAS: 70-47-3
L-Aspartic Acid	Sigma-Aldrich	Cat#A9256; CAS: 56-84-8
L-Cysteine hydrochloride monohydrate	Sigma-Aldrich	Cat#C7880; CAS: 7048-04-6
L-Glutamic Acid	Sigma-Aldrich	Cat#G1251; CAS: 56-86-0
L-Histidine	Sigma-Aldrich	Cat#H8000; CAS: 71-00-1
L-Isoleucine	Sigma-Aldrich	Cat#I2752; CAS: 73-32-5
L-Leucine	Sigma-Aldrich	Cat#L8000; CAS: 61-90-5
L-Lysine monohydrochloride	Sigma-Aldrich	Cat#L5626; CAS: 657-27-2
L-Methionine	Sigma-Aldrich	Cat#M9625; CAS: 63-68-3
L-Phenylalanine	Sigma-Aldrich	Cat#P2126; CAS: 63-91-2
L-Proline	Sigma-Aldrich	Cat#P0380; CAS: 147-85-3
L-Serine	Sigma-Aldrich	Cat#S4500; CAS: 56-45-1
L-Threonine	Sigma-Aldrich	Cat#T8625; CAS: 72-19-5
L-Tryptophan	Sigma-Aldrich	Cat#T0254; CAS: 73-22-3
L-Tyrosine	Sigma-Aldrich	Cat#T3754; CAS: 60-18-4
L-Valine	Sigma-Aldrich	Cat#V0500; CAS: 72-18-4
Latrunculin A	abcam	Cat#ab144290; CAS: 76343-93-6
Lithium acetate dihydrate	Sigma-Aldrich	Cat#L6883; CAS: 6108-17-4
Magnesium Chloride	Merck	Cat#105833; CAS: 7791-18-6
MangoMix PCR Master Mix	Bioline	Cat#BIO-25033
Methanol	Merck	Cat#106009; CAS: 67-56-1
*myo*-Inositol	Sigma-Aldrich	Cat#I7508; CAS: 87-89-8
Nourseothricin	Jena Bioscience	Cat#AB-102; CAS: 96736-11-7
*Pac*I	New England BioLabs	Cat#R054S
Peptone	BD Biocsciences	Cat#211677
Poly(ethylene glycol) 3350	Sigma-Aldrich	Cat#P3640; CAS: 25322-68-3
Potassium Acetate	Merck	Cat#104820; CAS: 127-08-2
SNAP-Surface Alexa Fluor 647	New England BioLabs	Cat#S9136S
Sodium Chloride	Merck	Cat#106404; CAS: 7647-14-5
ssDNA	Sigma-Aldrich	Cat#D1626; CAS: 438545-06-3
Sucrose	Sigma-Aldrich	Cat#S0389; CAS: 57-50-1
T4 DNA Ligase	ThermoFisher Scientific	Cat#EL0011
TetraSpeck beads (0.1 μm)	ThermoFisher Scientific	Cat#T7279
Triton X-100	Sigma-Aldrich	Cat#X100; CAS: 9002-93-1
Trizma base	Sigma-Aldrich	Cat#T1503; CAS: 77-86-1
Tryptone	BD Biocsciences	Cat#211705
Uracil	Sigma-Aldrich	Cat#U0750; CAS: 66-22-8
Velocity DNA Polymerase	Bioline	Cat#BIO-21098
Yeast Extract	BD Biocsciences	Cat#212750
Yeast Nitrogen Base w/o Amino Acids	BD Biocsciences	Cat#291940
α-D-Raffinose	Serva	Cat#34140; CAS: 17629-30-0

**Experimental Models: Organisms/Strains**		

*S. cerevisiae* MK0100	Kaksonen lab	N/A
*S. cerevisiae* MK0102	Kaksonen lab	N/A
A full list of strains is presented in Table S3	This paper	N/A

**Recombinant DNA**		

pFA6a-EGFP-HIS3MX6	Janke et al., 2004	N/A
pJR58 pFA6a-mMaple-HIS3MX6	This paper	N/A
pMM02 pFA6a-mMaple-hphNT1	This paper	N/A
pJR40 pFA6a-SNAPf-HIS3MX6	This paper	N/A
pMaM173	Khmelinskii et al., 2011	N/A
pMaM173-mMaple	This paper	N/A

**Software and Algorithms**		

Blender 2.78	Blender Foundation	https://www.blender.org/
Cytosim	Nédélec and Foethke, 2007	https://github.com/nedelec/cytosim
Fiji (ImageJ)	Schindelin et al., 2012	http://fiji.sc/
MATLAB	MathWorks	https://www.mathworks.com/products/matlab.html
Running Z-Projector Fiji plugin	Nico Stuurman	https://valelab4.ucsf.edu/∼nstuurman/IJplugins/Running_ZProjector.html
SMAP (Single Molecule Analysis Platform)	Ries lab	https://github.com/jries/SMAP
μManager	Edelstein et al., 2010	https://micro-manager.org/

**Other**		

160x NA 1.43 TIRF objective (HCX PL APO 160x/1.43 Oil CORR GSD)	Leica Microsystems	N/A
24 mm round glass coverslips (No. 1.5H)	Marienfeld	Cat#0117640
525/50 BrightLine single-band bandpass filter	Semrock	Cat#FF03-525/50-25
60x NA 1.49 TIRF objective	Nikon	N/A
640 LP dichroic mirror	Chroma	Cat#ZT640rdc
676/37 BrightLine single-band bandpass filter	Semrock	Cat#FF01-676/37-25
ET600/60 emission filter	Chroma	Cat#NC458462
Evolve512D EMCCD camera	Photometrics	N/A
iChrome MLE-L laser box (405, 488, 561, 638 nm)	Toptica Photonics	N/A
Ixon Ultra EMCCD camera	Andor	N/A
LightHUB laser box (405, 488, 561, 638 nm)	Omicron	N/A
Multimode fiber	Thorlabs	Cat#M105L02S-A
Piezo objective positioner	Physik Instrumente	N/A
PlasmaPrep2 plasma cleaner	Gala Instrumente	N/A
Transmissive laser speckle reducer	Optotune	Cat#LSR-3005-17S-VIS

### Contact for Reagent and Resource Sharing

Further information and requests for resources and reagents should be directed to and will be fulfilled by the Lead Contact, Jonas Ries (jonas.ries@embl.de).

### Experimental Model and Subject Details

All yeast strains used in this study were derivatives of *S. cerevisiae* MKY0100 or MKY0102 (Kaksonen lab). Construction of the strains is described below and a complete list of the strains used in this study is given in Table S3. Yeast cells were inoculated from single colonies on plates into 4 mL YPAD in a glass tube, and grown overnight at 30°C with shaking. The next morning, the culture was rediluted into 10 mL YPAD in a glass flask to OD_600_ of 0.25, and grown for 3-4 more hours at 30°C for sample preparation, typically reaching OD_600_ of 0.6-1.0. *SLA1* and *SLA2* deletion strains were incubated at 25°C and grown for longer times to reach the desired OD_600_, but otherwise prepared identically. For imaging of Latrunculin A arrested endocytic sites, cells were grown overnight in YPAD but diluted the next morning in SC-Trp to reduce the autofluorescent background for live cell experiments.

### Method Details

#### Yeast strain creation

Yeast strains expressing endocytic proteins tagged with mMaple (McEvoy et al., 2012) or SNAP_f_tag (Sun et al., 2011) at their C-termini were generated by homologous recombination using the PCR cassette system (Janke et al., 2004) in the parental strains MKY0100, MKY0102, MKY0122, MKY1596 and MKY2832. Sla2 was tagged with GFP in a parental strain expressing Ede1-mMaple. Correct integration of the tag was validated by colony PCR and fluorescence microscopy. Tagging plasmids containing the coding sequences of mMaple or SNAP_f_tag were created by replacing the coding sequence of GFP in either pFA6a-EGFP-HIS3MX6 (for histidine auxotrophy as selectable marker) or pFA6a-GFP-hphNT1 (for hygromycin resistance as selectable marker) between the *Sal*I and *Bam*HI restriction sites. The plasmid pMaM173-mMaple for N-terminal tagging was derived in a similar way from pMaM173 by replacing the parts specific for sfGFP using the SalI, *Nco*I, *Pac*I and BamHI restriction sites. Las17-mMaple was mated with MKY0764 to obtain the *SLA2* deletion with Las17-mMaple (JRY0040). JRY0038 was mated with MKY3247 to obtain the *BBC1* deletion in Las17-mMaple (JRY0076). The *SLA1* deletion with Las17-mMaple and Abp1-GFP (JRY0084) was generated by mating JRY0041 with MKY1596. For N-terminal tagging of Myo5, the protocol for seamless tagging was used (Khmelinskii et al., 2011). Here, a DNA cassette containing the first 180 bp of mMaple, a selectable marker for the synthesis of uracil surrounded by two I-*Sce*I restriction sites and full-length mMaple is inserted into MKY1743 using homologous recombination. After correct integration of this cassette, the expression of I-*Sce*I is induced by cultivation on plates containing galactose. This leads to an excision of the selectable marker and repair of the resulting double strand break using the fragment of mMaple and full-length mMaple as templates. Successful removal of the cassette is tested by negative selection against the URA3 marker using a 5-FOA plate.

#### Sample preparation for imaging

24 mm round glass coverslips were cleaned overnight in methanol/hydrochloric acid (50/50) while stirring. They were then rinsed repeatedly with water until the pH of the washing solution remained neutral. Subsequently, they were plasma cleaned for 5-10 min. A drop of 20 μL ConA solution (4 mg/mL in PBS) was added to each coverslip, and let incubate for 30 min in a sealed, humidified atmosphere to avoid evaporation. Then, the remaining liquid was removed and the coverslips were dried overnight at 37°C.

Prior to the day of imaging, yeast cells were inoculated from single colonies on plates into 4 mL YPAD in a glass tube, and grown overnight at 30°C with shaking. The next morning, the culture was rediluted into 10 mL YPAD in a glass flask to OD_600_ of 0.25, and grown for 3-4 more hours at 30°C for sample preparation, typically reaching OD_600_ of 0.6-1.0. *SLA1* and *SLA2* deletion strains were incubated at 25°C and grown for longer times to reach the desired OD_600_, but otherwise prepared identically.

Because endocytosis proceeds considerably faster than typical image acquisition times in SMLM, we chemically fixed the cells in order to obtain images with the highest possible spatial resolution (Mund et al., 2014). For this, 2 mL of the culture were collected by centrifugation at 500 *rcf.* for 2.5 min, resuspended in 100 μL YPAD and pipetted on a ConA coated coverslip, which has been briefly rinsed with water. The cells were allowed to settle for 15 min in a humidified atmosphere to prevent evaporation, and protected from light. After settling, the coverslip was directly transferred into the freshly prepared fixation solution containing 4% (w/v) formaldehyde, 2% (w/v) sucrose in PBS. Fixation was allowed to proceed for 15 min at gentle orbital shaking. The coverslip was then incubated for 15 min in 100 mM NH_4_Cl in PBS to quench remaining aldehyde groups. Quenching was repeated once more, before the coverslip was washed 3 times 5 min in PBS. At this point, the sample was ready for single color superresolution imaging.

For simultaneous dual-color imaging using mMaple and SNAP_f_tag (Figures 4A, 4C, and 5F), samples were processed further. 300 μL of permeabilization solution (0.25% (v/v) Triton X-100, 50% (v/v) ImageIT FX, in PBS) were added to the coverslip with cells facing up, and the coverslip was slowly agitated on an orbital shaker. The gentle shaking dissociated loosely bound cells from the coverslip, reducing the background. Permeabilization and blocking was allowed to proceed for 30 min, before the coverslip was washed 3 times 5 min in PBS. The coverslip was then transferred face down on a drop of 100 μL staining solution (1 μM SNAP Surface Alexa Fluor 647, 1% (w/v) BSA, 1 mM DTT, 0.25% (v/v) Triton X-100, in PBS) on parafilm. After staining for 2h, the sample was washed 3 times 5 min in PBS.

For the dual-color imaging of Ede1 and Sla2 (Figure 3E), GFP-tagged Sla2 was stained with nanobodies that are specific for GFP and are conjugated to Alexa Fluor 647 using sortase tagging. Yeast cells on a coverslip were fixed, permeabilized and blocked as described above. Subsequently, the coverslip was transferred face down on a drop of 100 μL nanobody staining solution (anti-GFP nanobody, 1% (w/v) BSA, 0.25% (v/v) Triton X-100, in PBS) on parafilm and incubated for 2 hr in the dark under a humidified atmosphere. After washing the coverslip 3 times for 5 min in PBS the sample was ready for simultaneous imaging of mMaple and Alexa Fluor 647.

For imaging of Latrunculin A arrested endocytic sites, cells were grown overnight in YPAD but diluted the next morning in SC-Trp to reduce the autofluorescent background for live cell experiments. For live cell imaging, 200 μL of a log phase culture were mixed with 2 μL of 20 mM LatA in DMSO (f.c. 200 μM) and pipetted onto a ConA coated coverslip. After 15 min of incubation to let the cells settle and LatA take effect, the medium was replaced by 200 μL fresh SC-Trp supplemented with 200 μM LatA to further reduce background fluorescence. The arrested endocytic patches were imaged until they clustered too much to distinguish individual sites, which was typically 45 min. As comparison a sample with fixed LatA arrested cells was prepared. For this, 1 mL from the same culture was spun down, resuspended in 100 μL of SC-Trp, supplemented with 200 μM LatA and pipetted onto a ConA coated coverslip. After 10 min, 100 μL of 8% (w/v) formaldehyde in SC-Trp was added onto the coverslip and incubated for another 10 min. Subsequently, the sample was prepared as described above for single color sample preparation by fixation, quenching, and washing.

#### Superresolution imaging

##### Single-color superresolution imaging

All single-color superresolution images were acquired on a custom-built, fully automated microscope, which was built for stable long-term automated image acquisition, and featured homogeneous high power illumination as described previously (Deschamps et al., 2016). The free-space output of a commercial LightHUB laser box with 405 nm, 488 nm, 561 nm and 638 nm laser lines was collimated, focused on a speckle reducer and coupled into a multimode fiber. The output of the fiber was then imaged into the sample to homogenously illuminate a circular area of ∼1000 μm^2^. Fluorescence was collected through a 160x NA 1.42 TIRF objective, filtered by a bandpass filter (for GFP: 525/50; for mMaple: 600/60), and focused onto an Evolve512D EMCCD camera. The z focus was optically stabilized by total internally reflecting an additional IR laser off the coverslip onto a quadrant photo diode, which was coupled into an electronic feedback loop with the piezo objective positioner. Z focus stability was typically better than 5 nm/h. All microscope components are controlled by a custom-written plugin for μManager (Edelstein et al., 2010).

For single-color superresolution imaging, samples were mounted in a D_2_O-based imaging buffer (50 mM Tris-HCl pH 8 in 95% D_2_O) to improve brightness (Ong et al., 2015). After selection of a region of interest, the back focal plane was imaged to ensure that the immersion oil contained no air bubbles. Then, videos of typically 10,000-100,000 frames were acquired using 561 nm illumination at ∼10 kW/cm^2^ in the specimen plane at 25 ms exposure times with an EM gain of 200. During the experiment, mMaple was sparsely photoconverted to its red state by 405 nm illumination, making sure that single non-overlapping PSFs were observed. Using an automated feedback loop, 405 nm laser power was adjusted to keep a constant number of localizations per frame throughout the experiment. When no more blinking was observed, the experiment was terminated.

##### Automated superresolution imaging

The sample was mounted in imaging buffer and subsequently sealed airtight using parafilm to prevent evaporation of the buffer. For each sample, we first focused on the midplane of yeast cells, and acquired several fields of view to get a qualitative impression about the number and distribution of endocytic sites in each strain. We then adjusted the focal plane to ∼300 nm above the coverslip in order to image endocytic sites at the bottom of the cells. Because endocytic invaginations grow perpendicular to the plasma membrane in yeast, with a small spread around the right angle (Kukulski et al., 2012, Picco et al., 2015), and our depth of field is more than 600 nm as determined from bead stacks, this allowed us to obtain two-dimensional projections of endocytic structures along their axis of invagination into the plane of the plasma membrane.

Using μManager, a grid of 100-500 positions was defined around the center of the stage, with each position spaced 200 μm away in every direction from the next region of interest to avoid cross-excitation and activation of neighboring positions due to scattered laser light. For each grid position, a set of acquisitions was performed automatically, each with a pre-defined set of settings for laser intensities, lens, and filter positions. First, a blinking video was recorded as described above, which was automatically stopped once the 405 nm intensity reached a threshold value, indicating that all mMaple molecules have been imaged and bleached. Subsequently, an image of the back focal plane was recorded to check for air bubbles in the immersion oil.

For some experiments, mMaple superresolution imaging was combined with diffraction-limited GFP imaging to obtain an additional, diffraction-limited reference signal. GFP has been previously shown to have no substantial cross-talk into the mMaple channel (Puchner et al., 2013). For this, z stacks of ± 2 μm were recorded in 50 nm steps around the focal plane in the GFP channel after mMaple superresolution imaging was completed. Once all different types of acquisitions were completed for one grid position, the stage was moved to the next one. In between different positions, a waiting time of 15 s allowed for mechanical equilibration after stage movement. The measurement cycle was repeated until all positions were imaged, or the experiment was terminated manually.

##### Dual-color imaging

The dual-color images shown in Figures 4 and 5 were acquired on a custom-built microscope with single-mode fiber-based illumination, where the single-mode output of a commercial iChrome MLE laser box with 405 nm, 488 nm, 561 nm and 638 nm laser lines was collimated, focused on the back focal plane of a 60 x NA 1.49 TIRF objective, and adjusted for epi illumination. Fluorescence emission was laterally constricted by a slit, split using a 640 LP dichroic mirror, separately filtered by 600/60 (mMaple signal) and 676/37 (Alexa Fluor 647 signal) bandpass filters, and imaged on two parts of the camera chip of an Ixon Ultra EMCCD camera. Analogous to the setup described above, the z focus was optically stabilized.

Samples were mounted in thiol-containing blinking buffer with an enzymatic oxygen scavenger (50 mM Tris pH 8, 10 mM NaCl, 10% (w/v) D-glucose, 35 mM cysteamine, 0.5 mg/mL glucose oxidase, 40 μg/mL catalase, in ∼90% D_2_O). After selection of a region of interest, the back focal plane was imaged to ensure that the immersion oil contained no air bubbles. Then, the sample was illuminated both with 561 nm and 640 nm light, and videos of typically 10,000-100,000 frames were acquired at 30 ms exposure times with an EM gain of 200. During the experiment, 405 nm laser intensity was automatically adjusted to keep a constant number of non-overlapping PSFs as described above. When no more blinking was observed, the experiment was terminated.

Dual-color images shown in Figure 3E were acquired on the microscope described under *Single-color superresolution imaging* with the following modifications: The signal of the 2 fluorophores was split using a 640 LP dichroic mirror, separately filtered by 600/60 and 676/37 bandpass filters, and imaged on two parts of the camera chip.

#### Data analysis

All data analysis was performed using a custom comprehensive analysis software framework, SMAP (“Superresolution Microscopy Analysis Platform”, unpublished data), which was developed in MATLAB.

##### Single molecule localization

For localization, peaks were detected in the raw images by smoothing, background subtraction using a wavelet filter, and non-maximum suppression. Peaks with intensities above a dynamically determined threshold were then localized by fitting a pixelated Gaussian function with a homogeneous photon background. Fitting of individual PSFs was highly parallelized by using a GPU-based algorithm of a maximum-likelihood estimator for data that is Poisson distributed (Smith et al., 2010).

All experiments were automatically fitted online during the acquisition. During automatic acquisitions, new experiments were automatically detected and subjected to localization.

##### Image reconstruction

All image reconstruction was done in SMAP. Localizations that were found in consecutive frames (a gap of 1 frame was allowed) within a circular range of 75 nm radius were grouped into a single localization.

Localizations with a localization precision worse than 30 nm and a fitted PSF standard deviation larger than 175 nm were discarded. These cutoffs are loose, and retained the majority of localizations, but efficiently removed dim localizations resulting from autofluorescent background and out-of-focus events.

All filtered localizations were plotted at their coordinates as normalized Gaussians with a standard deviation proportional to their localization precision. To increase visibility of very precisely localized events, a minimum Gaussian standard deviation of 6 nm was used. To equalize pixels with very high brightness, the contrast was adjusted to saturate 0.01%–0.1% of the brightest intensity values.

To correct for sample drift during the image acquisition, localizations were sorted according to the frame in which they were detected and binned in ten time windows, for which individual superresolution images were calculated. Then, the pairwise image cross-correlation of all intermediate images with all others was calculated, and spline interpolation was used to calculate the lateral drift trajectory, which was then corrected for. Typically, we observed a lateral drift of 20-100 nm/h. Drift correction was not applied for very short experiments with less than 5000 frames or when the detected lateral drift was below 10 nm.

##### Dual-color image reconstruction

In our dual-color imaging, the signals from mMaple and Alexa Fluor 647 were imaged on two separate parts of the camera chip. To overlay both channels, we experimentally determined a transformation function using a calibration bead sample. For the bead sample, we diluted TetraSpeck beads 1:200 in 100 mM MgCl_2_ on a glass coverslip. We imaged at least 1,000 beads in both channels, localized them, and calculated a projective transformation from their positions. The accuracy of this transformation was better than 10 nm, as judged by the standard deviation of individual bead positions. This transformation was subsequently used to transform the mMaple channel onto the Alexa Fluor 647 channel.

#### Analysis of endocytic structures

##### Quality control of automated imaging

Datasets that were generated by automatic superresolution imaging typically consisted of 100 to 500 fields of view, and were first subjected to quality control. All individual back focal plane (BFP) images were inspected for air bubbles. If single BFPs showed bubbles, the corresponding fields of view were discarded. If multiple BFPs showed bubbles, the entire dataset was discarded.

Subsequently, statistics from the individual superresolved images were compared. We analyzed the median localization precision, median fitted photon background, total number of localizations and number of frames for each experiment. If any of those parameters did not remain approximately constant over time, this indicated changes in the sample or imaging conditions, e.g., a change in salt concentration or pH due to buffer evaporation. In this case, the experiment was either entirely discarded, or only the first adequate fields of view were used.

##### Segmentation of cells and endocytic sites

Cells were segmented in the superresolved images by filtering the image with a large Gaussian blur, and detecting peaks above a user-defined threshold, which was adjusted so that all cells were segmented properly.

To segment endocytic sites, the superresolved image of the cell was rendered with a pixel size of 200 nm and masked using a user-defined threshold, which was adjusted once for each endocytic protein corresponding to their different abundances. This mask roughly represented the boundary of the cell. Then, a convex hull was drawn around all localizations within this mask, and subsequently constricted by iteratively removing the points on the hull 3 times. The resulting convex hull faithfully enveloped the cellular signal. The convex hull was then shrunk by at least 30% to define the center bottom of the cell, where endocytic sites were then segmented as follows.

To segment endocytic sites, the superresolved image was rendered with a pixel size of 100 nm. In the filtered image, peaks above a user-defined threshold were picked. This cutoff was adjusted once for each endocytic protein. To avoid closely juxtaposed sites an upper size limit was empirically set, and visually confirmed to not be too restrictive and to only exclude clear double-sites, and no big individual sites. The size range was adjusted once for each protein.

For clathrin (Clc1 and Chc1), site segmentation was complicated by the fact that in yeast a major fraction of clathrin molecules are found at intracellular membrane compartments, which are often close to the plasma membrane. Thus, we cannot rule out that a minor part of the segmented structures are not actually endocytic sites, but comparably small intracellular compartments, which might confound the structural analysis.

Endocytic sites in *SLA1* and *SLA2* deletion strains, in LatA arrested live cell experiments as well as in dual color experiments were picked manually.

##### Geometric analysis of endocytic sites

Superresolved images of individual endocytic sites were rendered with a pixel size of 3 nm, and fitted with a geometric model that describes the shape either as ring or as patch, where rings have a hole in their center, around which there is a rim of a certain thickness. Patches do not have holes. Both rings and patches are described by their outer radius *r*_*out*_ and the rim thickness *dr*. Because of their central holes, rings have *r*_*out*_ *> dr*, whereas patches have *r*_*out*_ *≤ dr*. The model is illustrated below and formally described in Equations 1 and 2, where *I(X, Y)* is the pixelated image, *x*_*0*_*, y*_*0*_ are the coordinates of the center, *r*_*out*_ is the outer radius, *dr* is the rim thickness, and *A* is a scaling factor. To account for the expected localization precision, a Gaussian blur with σ = 15 nm is introduced.

(1) $$f\left(X,Y\right)=A\left(\text{erf}\left(\frac{r_{out}-R\left(X,Y\right)}{\sqrt{2}\sigma }\right)-\text{erf}\left(\frac{r_{out}-dr-R\left(X,Y\right)}{\sqrt{2}\sigma }\right)\right)$$

(2) $$R\left(X,Y\right)=\sqrt{{\left(X-x_{0}\right)}^{2}+{\left(Y-y_{0}\right)}^{2}}$$

(3) $$\left\{dr,r_{out},x_{0},y_{0}\right\}=\underset{dr,r_{out},x_{0},y_{0}}{\text{argmin}}\left(\sum_{X,Y}{\left(I\left(X,Y\right)-f\left(X,Y\right)\right)}^{2}\right)$$

Least-squares fitting was then performed numerically as shown in Equation 3. We then calculated the radial distribution of the localizations around the fitted center coordinates to obtain the radial profile of individual endocytic sites.

As we used a low threshold during automatic site segmentation to avoid biases from overly stringent segmentation, the dataset contained a comparably small set of structures with a low number of localizations and a small size on the order of the resolution. Because these structures were too small to obtain geometric information, we excluded structures with outer radii below 30 nm and below 30 localizations, i.e., with less than approximately 10 mMaple proteins, from the analysis. Because structures in the coat and scission modules (Clc1, Chc1, End3, Ent1, Pan1, Rvs167, Sla1, Sla2) mostly formed small structures, we only excluded structures with less than 30 localizations, and did not apply a size threshold. We then aligned the superresolution images and radial profiles of all endocytic sites by their center coordinates, and calculated the average images and radial profiles.

Proteins of the actin module (Abp1, Arc18, Cap1, Cap2, Crn1, Sac6, Twf1) on average assembled into large structures (outer radius > 80 nm) with a slight minimum in the center of their radial profiles (density at the center is more than 80% of the maximum intensity). Because our images acquired in the equatorial plane (Figure 5) show that the actin network occupies a large, hemispherical volume around the endocytic site, which agrees with previous reports using electron microscopy (Kukulski et al., 2012), we classified these structures as dome-shaped.

For dual-color bottom-view images (Figures 3 and 4), both colors were analyzed analogously. In the geometric fit, both structures were fitted with common center coordinates (Figure 4), or aligned by the fitted center coordinates of Sla2 (Figure 3).

To quantify GFP intensity corresponding to superresolved endocytic sites (Figures 3, 4, and 5), we first calculated a maximum intensity projection of the 7 z stack slices around the focal plane. The projected images were corrected for chromatic aberrations between the GFP and mMaple channels using a transformation that was pre-determined using TetraSpeck beads, as described above. The background was calculated using a wavelet filter of level 3, and subtracted from the transformed images. For each endocytic site, the corresponding GFP intensity was determined by fitting the GFP image with three Gaussians: a first one fixed at the center coordinate of the superresolved site, and a second and third one with variable centers at least 350 nm away from the first one and each other to account for potential partial overlap of the diffraction-limited signals from proximal sites, which occurred frequently. If a site was entirely isolated, the amplitude of the second and third Gaussians were negligibly low. To avoid artificially high fitted GFP intensities that result from overlapping GFP signals from more than 2 sites, as was the case mostly in small buds, we excluded endocytic sites from the analysis where the fitted GFP intensity typically was more than 1.5 times the 80^th^ percentile of GFP intensities, which we empirically determined to faithfully exclude the highest GFP intensities. Eventually, we binned sites by their corresponding normalized GFP intensities into a bin with “no GFP” containing all sites with a normalized intensity < 0.1 times the 80^th^ percentile of GFP intensities, and three same-sized bins “low GFP,” “medium GFP” and “high GFP” containing equal share of the remaining sites sorted by GFP intensity.

##### Analysis of dual-color side-view images

Endocytic sites were segmented and rotated manually so that the direction of endocytosis is oriented upward. Then, the structures from both channels were segmented by thresholding and masking. For both channels, localizations were projected on the axis of endocytosis, and centroid and quantiles of the distribution along this axis were calculated. Endocytic sites were then aligned at the bottom by the 5^th^ percentile of the Las17 localizations, laterally aligned manually, and sorted by increasing Abp1 centroid position. The dual color images were rendered, and running window averages were calculated in Fiji using the Running Z-Projector plugin using ‘Average intensity’ and ‘window size 7’. We then compared the resulting averages to time-resolved outer boundaries of the actin network, that were determined as ribosome exclusion zones by CLEM (Kukulski et al., 2012). Using the centroid distances from Picco et al. (2015), we determined that the first of the running window averages corresponds approximately to −7 s, and that the last average corresponds to approximately 1 s. We assumed that the remaining frames are equally spaced in between, and manually overlaid the respective exclusion zone shapes from Figure 7 of Kukulski et al. (2012), using the bottom of Las17 to align the membrane.

#### Simulation of actin polymerization in Cytosim

In brief, the Brownian dynamics simulation contains actin filaments assembled into a 3D network producing forces on the membrane. Filaments are nucleated by Arp2/3 complexes and connected by crosslinkers. The membrane invagination is represented by a movable object of constant shape, to which some actin filaments are bound via connectors mimicking Sla2/Ent1 proteins. All objects diffuse within a cylindrical volume built around an active patch on the plasma membrane, and may associate stochastically upon collision. The mechanical equilibrium of the network is simulated using overdamped Langevin dynamics (Nédélec and Foethke, 2007), thus including Brownian motion and elasticity of the network. Chemical association/dissociations between the components, and filament assembly, are simulated stochastically following a modified Gillespie algorithm, which includes the effect that forces may have on the reaction rates. We describe the elements and methods of simulations in more details below.

##### Filament mechanics

Actin is modeled as slender elastic beams of rigidity κ, hence with a persistence length *l*_*p*_ *= κ / k*_*B*_ *T*, where *k*_*B*_ *T* is the thermal energy, κ = *0.08* pNμm^2^ (Gittes et al., 1993) and thus *l*_*p*_ *≈20* μm. The filaments are represented as strings of points separated by a distance *s = 5.5* nm, and bending rigidity promotes the alignment of these points (Nédélec and Foethke, 2007). For simplicity, actin filaments are not helical, i.e., they are cylindrical with complete rotational symmetry around their central axis, although they are displayed as helices on the figures for increased realism.

##### Actin steric interactions

The model includes steric interaction between actin filaments: two filaments located at a distance *d* repel each other if *d<h*, with a force *k*_*s*_*(d-h)*, where *h = 8* nm and *k*_*s*_ *= 20,000* pN/μm. This repulsive force is soft, and extends slightly further than the atomic radius of F-actin of *4.5* nm, as it is meant to represent the electrostatic interactions between filaments, as well as between proteins bound to the filaments, rather than solely the physical radius of actin itself.

##### Actin confinement

Filaments in Cytosim are confined inside a volume *V* by adding a force *k*_*c*_ *(p-x)* to every model point *x* located outside *V*, where *p* is the projection of *x* on the edge of *V*. By convention in this work, the cell wall (and the membrane before invagination) is located at *z = 0*, and we used therefore *V = { (x,y,z) | z ≥ 0 }*. A filament pushing on the cell wall must have some of its model points with *z<0*, and these points receive a force *f = -k*_*c*_ *z* directed upward. We used a stiffness *k*_*c*_ *= 50,000* pN/μm, to match the mechanical characteristics of the cell wall in yeast cells, with a published Young Modulus *Y ∼120* MPa (Smith et al., 2000a, Smith et al., 2000b). The simulation volume was big enough that actin was unconstrained laterally and at the top.

##### Actin dynamics

We assumed that actin filaments assemble only at the barbed end, and implemented a Brownian ratchet model as previously described (Hill, 1981). Assuming a concentration *A*_*1*_ of free actin monomers, the polymerization rate *k*_*+*_ and depolymerization rate *k*_*-*_ for a free actin end is:

$$k_{+}=k_{on}\;A_{1},\;k_{-}=k_{off}$$

such that *k*_*off*_ /*k*_*on*_ = *A*_*1*_^∗^ is the critical G-actin concentration.

The Brownian ratchet model predicts the effect of a force *f ≥ 0* opposing actin assembly:

$$k_{+}=k_{on}\;A_{1}e^{-f\delta /k_{B}T},\;k_{-}=k_{off}$$

in which *δ ≈2.8* nm is the extension provided by the addition of one monomer. From there, we can define *f*_*0*_ *= k*_*B*_ *T* log*(A*_*1*_*/A*_*1*_*^∗^) / δ* as the stall force of actin assembly (Hill, 1981). Using *k*_*on*_ = *400* s^-1^, *k*_*off*_ = *1.4* s^-1^ and the free actin concentration measured *in vivo* in fission yeast *A*_*1*_ *= 40* μM (Wu and Pollard, 2005), one finds *f*_*0*_ *∼9* pN (Dmitrieff and Nédélec, 2016).

This force is greater than the force measured *in vitro* (Kovar and Pollard, 2004), but much lower concentrations of G-actin were used in these experiments, and indeed the stall force depends critically on the concentration of free monomers (*A*_*1*_) such that higher forces are possible *in vivo*.

Actin filaments reaching a length of 60 nm are capped, cease growing and remain at this length.

##### Actin nucleation

In yeast endocytosis, the core nucleation activity of Arp2/3 is activated by Las17. In the simulation, we simplified this process by assuming that Arp2/3 is rapidly activated by Las17, and rapidly inactivated once Las17 is released. With these assumptions, Arp2/3 is effectively active only in the “nucleation zone” defined by the localization of Las17. This nucleation zone on the plasma membrane (*z = 0*) is represented in the simulation as a ring (*R*_*i*_^*2*^ *< x*^*2*^ *+ y*^*2*^ *< R*_*o*_^*2*^ ). We used *R*_*i*_ *= 10* nm and *R*_*o*_ *= 55* nm, slightly smaller than the measured outer radius of Las17 of ∼70 nm. This compensates for the current limitation of Cytosim that prevented us from implementing the true radial density profile of Las17, but restricted us to implementing a simple ring shape where Las17 is homogenously distributed.

We modeled the gradual build-up of active Arp2/3 after *in vivo* data: after an initial phase of slow Arp2/3 accumulation (*k*_*a*_^*0*^ *= 5 s*^*-1*^), the rate of Arp2/3 addition increases eightfold (*k*_*a*_^*1*^ *= 40 s*^*-1*^) (Picco et al., 2015). For simplicity, we did not include the biochemical feedbacks that could underlie this simple constant accumulation of Arp2/3 (Wang et al., 2016).

Arp2/3 is modeled as a complex including a binder and a nucleator. The binder may bind to any filament at any location, and never detaches from this filament. The nucleator is only active if the binder is attached, and in this case creates filaments with a rate *k*_*nuc*_.

The nucleator stays attached to a new actin filament that it nucleated, and remains inactive henceforth. The number of filaments therefore cannot exceed the number of nucleators.

Mechanically, Arp2/3 entities that are bound to two filaments behave as stiff corner brace with a resting angle of 70 degrees, and angular stiffness of *k*_*θ*_ *= 1 pN* μm/rad and a linear stiffness of *20,000* pN/μm.

Because this is the only mode of nucleation in the simulation, the network that forms is a single connected “tree,” and with this value of *k*_*θ*_, branch angles are close to 70 degrees.

##### Actin crosslinking

Crosslinkers in the endocytic machinery are bivalent complexes composed of two identical passive actin binders. Binders may attach with a rate *k*_*on*_ = 10/sec to any filament located within 18 nm, at the closest position, and crosslinkers may thus connect parallel and antiparallel filaments equally. Binders remain at fixed positions and can unbind stochastically following Kramer’s law:

$$k_{u}=k_{off}\;e^{f/f_{u}}.$$

Here *f*_*u*_ is the unbinding force, *k*_*off*_ the unbinding rate in the absence of force, and *k*_*u*_ the effective unbinding rate. We used *k*_*off*_ = 0.025 s^-1^ and *f*_*u*_ = 10 pN.

Mechanically, a crosslinker bound to two filaments behaves as a spring of resting length *l*_*c*_ *= 10* nm and stiffness *k*_*x*_ *= 5,000* pN/μm.

##### Actin-membrane connectors

In yeast cells, the endocytic actin network is connected to the membrane via a large number of Sla2 proteins, but their biochemical properties are unknown.

For this model, it appeared sufficient to use a simple approach in which actin is connected to the vesicle by a tether constituted of only three permanent binders, but the stiffness of these binder was set appropriately high *k*_*v*_ *= 200,000* pN/μm to represent multiple molecular connections. For simplicity, their resting length is zero and they never detach. As the simulation starts, these binders are unattached.

##### Invagination

The remodeling of the membrane by endocytosis involves the interplay between mechanical properties of the coated membrane and the forces generated by the actin cytoskeleton. We have previously studied the mechanics of the membrane while simplifying the actin cytoskeleton to the bare force that it is able to generate (Dmitrieff and Nédélec, 2016). We proceed inversely here, and simplify the membrane mechanics to focus on the actin cytoskeleton. The invagination is represented by an undeformable “cylinder” starting below the plasma membrane, with just three actin-connectors at its tip protruding inside the cell, representing the activity of the Sla2 patch.

To pull the invagination inward, actin has to overcome turgor pressure and membrane rigidity. Based on our previous estimate (Dmitrieff and Nédélec, 2015), we approximate the force by which the invagination resists, as a function of *L*, the length of the invagination (with *L = 0* corresponding to a flat membrane):

$$f\left(L\right)=\{\begin{array}{c} 0\;if\;L\leq 0\\ f_{0}+k_{\text{Π}}\;L\;if\;0<L<L_{0}\;\\ 0\;if\;L_{0}\leq L \end{array}.$$

This simplified dependence keeps the most important features of the force profile identified previously (Dmitrieff and Nédélec, 2015): a non-zero force *f*_*0*_ is needed to initially lift the membrane; the force increases steadily until a length *L*_*0*_, and vanishes above. This is characteristic of the “snap-through” transition identified in yeast endocytosis (Walani et al., 2015). Based on the values obtained by fitting the experimental invagination profiles (Kukulski et al., 2012), we used here *f*_*0*_ *= 200* pN, *L*_*0*_ = 60 nm and k_Π_ such that the force peaks at *f*_*0*_ + k_Π_ *L*_*0*_ = 1000 pN (Dmitrieff and Nédélec, 2015).

Endocytosis was considered successful if the invagination reached the distance *L*_*0*_ at which the snap-through transition occurs.

##### Radial distribution of proteins

To obtain the simulated radial distribution of proteins, we monitored the position of simulation elements in 382 centered simulations. When mentioned, we simulated localization precision by adding a normally-distributed random term on the x and y position, with a deviation of 15 nm per axis, i.e., an overall spread of 21.2 nm.

### Quantification and Statistical Analysis

#### Analysis of endocytic sites

For the different superresolution experiments at least two biological replicates were performed, in each of which typically images of 1,000-10,000 cells were acquired. In the individual experiments single fields of view were excluded as described in *Quality control of automated superresolution imaging* or entire experiments were repeated if quality standards were not met.

To statistically analyze the differences between the outer radii of two datasets, the null hypothesis of equal medians was tested using a Mann-Whitney U test (Wilcoxon rank-sum test, MATLAB function *ranksum*), which is non-parametric and does not rely on the data following any specific distribution. The definition of the different levels of significance is stated in the figure legends. n denotes the number of analyzed individual endocytic sites and can be found in the figures, the figure legends, the main text or Table S1.

#### Analysis of Cytosim simulations

Hundreds of simulations with the same parameters were run and analyzed, N denotes the numbers of simulations and is indicated in the figures. Their outcomes differ because most events in the simulation are stochastic (only the seed of the random number generator is changed).

### Data and Software Availability

The collection of MATLAB scripts used to reconstruct the superresolution images and to analyze the endocytic sites are available online (https://github.com/jries/SMAP).
