# Supplementary material for: Systematic Nanoscale Analysis of Endocytosis Links Efficient Vesicle Formation to Patterned Actin Nucleation
Source: Cell. 2018 Aug 9;174(4):884–896.e17. doi: 10.1016/j.cell.2018.06.032 (PMC6086932; doi:10.1016/j.cell.2018.06.032)
Supplement: Document S1. Tables S1–S3 [file mmc1.pdf]

Cell, Volume *174*

Supplemental Information

Systematic Nanoscale Analysis of Endocytosis Links Efficient Vesicle Formation to Patterned Actin Nucleation

Markus Mund, Johannes Albertus van der Beek, Joran Deschamps, Serge Dmitrieff, Philipp Hoess, Jooske Louise Monster, Andrea Picco, François Nédélec, Marko Kaksonen, and Jonas Ries

Table S1: Statistics of imaged endocytic sites, related to Figures 1, 2

Protein	Number of sites	Outer radius (fit) Mean ± SD (nm)	% rings (fit)	Average shape classification
Abp1	*2849*	*85 ± 21*	*32*	*Dome*
Arc18	*2554*	*83 ± 19*	*46*	*Dome*
Bbc1	*1236*	*98 ± 38*	*81*	*Ring*
Bzz1	*3030*	*69 ± 30*	*74*	*Ring*
Cap1	*4307*	*99 ± 29*	*50*	*Dome*
Cap2	*2719*	*96 ± 30*	*44*	*Dome*
Chc1	*1109*	*34 ± 28*	*22*	*Patch*
Clc1	*1167*	*25 ± 20*	*20*	*Patch*
Crn1	*3896*	*90 ± 26*	*44*	*Dome*
Ede1	*14219*	*66 ± 30*	*54*	*Ring*
End3	*6918*	*36 ± 18*	*18*	*Patch*
Ent1	*7087*	*40 ± 32*	*44*	*Patch*
Las17	*8599*	*70 ± 32*	*75*	*Ring*
Myo3	*6966*	*91 ± 45*	*85*	*Ring*
Myo5	*3663*	*92 ± 28*	*79*	*Ring*
Myo5 N-term	*1724*	*78 ± 31*	*80*	*Ring*
Pan1	*3376*	*47 ± 22*	*30*	*Patch*
Rvs167	*413*	*42 ± 16*	*35*	*Patch*
Sac6	*1378*	*89 ± 20*	*47*	*Dome*
Sla1	*5983*	*44 ± 27*	*19*	*Patch*
Sla2	*7367*	*40 ± 32*	*27*	*Patch*
Syp1	*3347*	*66 ± 31*	*66*	*Ring*
Twf1	*2771*	*101 ± 31*	*61*	*Dome*
Vrp1	*5455*	*70 ± 33*	*67*	*Ring*

For each of the 23 imaged endocytic proteins, the number of imaged sites, outer radii as obtained by the fit, fraction of ring-shaped sizes as indicated by the fit, and shape classification are shown. For details see STAR Methods.

Table S2: Statistics of endocytic proteins with GFP timing markers, related to Figures 3, 4, 5

Protein		Number of sites	Outer radius (fit) Mean ± SD (nm)
Ede1	no Sla2-GFP	*2514*	*61 ± 21*
	low Sla2-GFP	*2634*	*63 ± 19*
	med Sla2-GFP	*2634*	*67 ± 20*
	high Sla2-GFP	*2634*	*70 ± 22*
Pan1	no Abp1-GFP	*494*	*42 ± 20*
	low Abp1-GFP	*98*	*49 ± 23*
	med Abp1-GFP	*98*	*47 ± 17*
	high Abp1-GFP	*98*	*50 ± 16*
Las17 wt	no Abp1-GFP	*2550*	*67 ± 23*
	low Abp1-GFP	*1595*	*69 ± 24*
	med Abp1-GFP	*1595*	*70 ± 26*
	high Abp1-GFP	*1595*	*70 ± 27*
Las17 *bbc1*Δ	no Abp1-GFP	*1237*	*67 ± 22*
	low Abp1-GFP	*503*	*74 ± 30*
	med Abp1-GFP	*503*	*76 ± 32*
	high Abp1-GFP	*503*	*82 ± 31*
Las17 *sla1*Δ	no Abp1-GFP	*131*	*70 ± 20*
	low Abp1-GFP	*28*	*71 ± 24*
	med Abp1-GFP	*28*	*66 ± 18*
	high Abp1-GFP	*28*	*72 ± 21*
Abp1	no Rvs167-GFP	*1044*	*79 ± 27*
	low Rvs167-GFP	*568*	*80 ± 23*
	med Rvs167-GFP	*568*	*85 ± 21*
	high Rvs167-GFP	*568*	*88 ± 20*

Table S3: Yeast strains used in this study, related to STAR methods

Strain	Genotype	Source
MKY0100	MATa, his3Δ200, leu2-3,112, ura3-52, lys2-801	Kaksonen lab
MKY0102	MATα, his3Δ200, leu2-3,112, ura3-52, lys2-801	Kaksonen lab
MKY0122	MATa, his3Δ200, leu2-3,112, ura3-52, lys2-801, ABP1-GFP::HIS3MX6	Kaksonen lab
MKY0764	MATa his3Δ200, leu2-3,112, ura3-52, lys2-801 sla2Δ::natNT2	Kaksonen lab
MKY1596	MATα, his3Δ200, leu2-3,112, ura3-52, lys2-801, sla1Δ::natNT2	Kaksonen lab
MKY1743	MATa, his3Δ200, leu2-3,112::natNT2-GAL1pr-I-SceI, ura3-52, lys2-801	Kaksonen lab
MKY2832	MATa, his3Δ200, leu2-3,112, ura3-52, lys2-801, RVS167-GFP::HIS3MX6	Kaksonen lab
MKY3247	MATa, his3Δ200, leu2-3,112, ura3-52, lys2-801, ABP1-EGFP::HIS3MX6, bbc1Δ::natNT2	Kaksonen lab
JRY0009	MATα, his3Δ200, leu2-3,112, ura3-52, lys2-801, ABP1-mMaple::HIS3MX6	This study
JRY0014	MATa, his3Δ200, leu2-3,112, ura3-52, lys2-801, LAS17-SNAP::HIS3MX6, ABP1-mMaple::hphNT1	This study
JRY0020	MATα, his3Δ200, leu2-3,112, ura3-52, lys2-801, ARC18-mMaple::HIS3MX6	This study
JRY0023	MATα, his3Δ200, leu2-3,112, ura3-52, lys2-801, BZZ1-mMaple::HIS3MX6	This study
JRY0024	MATα, his3Δ200, leu2-3,112, ura3-52, lys2-801, CAP1-mMaple::HIS3MX6	This study
JRY0027	MATα, his3Δ200, leu2-3,112, ura3-52, lys2-801, CLC1-mMaple::HIS3MX6	This study
JRY0030	MATα, his3Δ200, leu2-3,112, ura3-52, lys2-801, EDE1-mMaple::hphNT1	This study
JRY0032	MATa, his3Δ200, leu2-3,112, ura3-52, lys2-801, LAS17-SNAP::HIS3MX6, SLA2-mMaple::hphNT1	This study
JRY0033	MATa, his3Δ200, leu2-3,112, ura3-52, lys2-801, MYO5-SNAP::HIS3MX6, LAS17-mMaple::hphNT1	This study
JRY0034	MATα, his3Δ200, leu2-3,112, ura3-52, lys2-801, SLA2-GFP::HIS3MX6, EDE1 mMaple::hphNT1	This study
JRY0035	MATα, his3Δ200, leu2-3,112, ura3-52, lys2-801, END3-mMaple::HIS3MX6	This study
JRY0037	MATα, his3Δ200, leu2-3,112, ura3-52, lys2-801, ENT1-mMaple::HIS3MX6	This study
JRY0038	MATα, his3Δ200, leu2-3,112, ura3-52, lys2-801, LAS17-mMaple::HIS3MX6	This study
JRY0040	MATα, his3Δ200, leu2-3,112, ura3-52, lys2-801, LAS17-mMaple::HIS3MX6, sla2Δ::natNT2	This study
JRY0041	MATa, his3Δ200, leu2-3,112, ura3-52, lys2-801, ABP1-GFP::HIS3MX6, LAS17-mMaple::hphNT1	This study
JRY0046	MATα, his3Δ200, leu2-3,112, ura3-52, lys2-801, MYO5-mMaple::HIS3MX6	This study
JRY0058	MATα, his3Δ200, leu2-3,112, ura3-52, lys2-801, PAN1-mMaple::HIS3MX6	This study
JRY0059	MATα, his3Δ200, leu2-3,112, ura3-52, lys2-801, RVS167-mMaple::HIS3MX6	This study
JRY0061	MATα, his3Δ200, leu2-3,112, ura3-52, lys2-801, SAC6-mMaple::HIS3MX6	This study
JRY0064	MATα, his3Δ200, leu2-3,112, ura3-52, lys2-801, SLA1-mMaple::HIS3MX6	This study
JRY0065	MATα, his3Δ200, leu2-3,112, ura3-52, lys2-801, SLA2-mMaple::HIS3MX6	This study
JRY0069	MATα, his3Δ200, leu2-3,112, ura3-52, lys2-801, SYP1-mMaple::hphNT1	This study
JRY0071	MATα, his3Δ200, leu2-3,112, ura3-52, lys2-801, VRP1-mMaple::HIS3MX6	This study
JRY0072	MATa, his3Δ200, leu2-3,112, ura3-52, lys2-801, BBC1-mMaple::HIS3MX6	This study
JRY0073	MATa, his3Δ200, leu2-3,112, ura3-52, lys2-801, CAP2-mMaple::HIS3MX6	This study
JRY0074	MATa, his3Δ200, leu2-3,112, ura3-52, lys2-801, CHC1-mMaple::HIS3MX6	This study
JRY0075	MATa, his3Δ200, leu2-3,112, ura3-52, lys2-801, CRN1-mMaple::HIS3MX6	This study
JRY0076	MATα, his3Δ200, leu2-3,112, ura3-52, lys2-801, ABP1-EGFP::HIS3MX6, LAS17-mMaple::hphNT1, bbc1Δ::natNT2	This study
JRY0077	MATa, his3Δ200, leu2-3,112, ura3-52, lys2-801, MYO3-mMaple::HIS3MX6	This study
JRY0078	MATa, his3Δ200, leu2-3,112, ura3-52, lys2-801, TWF1-mMaple::HIS3MX6	This study
JRY0079	MATa, his3Δ200, GalL-ISce1-nat::leu2-3,112, ura3-52, lys2-801 mMaple-Myo5	This study
JRY0080	MATa, his3Δ200, leu2-3,112, ura3-52, lys2-801, ABP1-GFP::HIS3MX6, SLA1 mMaple::hphNT1	This study
JRY0081	MATa, his3Δ200, leu2-3,112, ura3-52, lys2-801, ABP1-GFP::HIS3MX6, PAN1 mMaple::hphNT1	This study
JRY0082	MATa, his3Δ200, leu2-3,112, ura3-52, lys2-801, RVS167-GFP::HIS3MX6, PAN1-mMaple::hphNT1	This study
JRY0083	MATa, his3Δ200, leu2-3,112, ura3-52, lys2-801, RVS167-GFP::HIS3MX6, ABP1-mMaple::hphNT1	This study
JRY0084	MATα, his3Δ200, leu2-3,112, ura3-52, lys2-801, ABP1-GFP::HIS3MX6, LAS17-mMaple::hphNT1, sla1Δ::natNT2	This study
